# Biostimulants in Viticulture: A Sustainable Approach against Biotic and Abiotic Stresses

**DOI:** 10.3390/plants11020162

**Published:** 2022-01-07

**Authors:** Eleonora Cataldo, Maddalena Fucile, Giovan Battista Mattii

**Affiliations:** Department of Agriculture, Food, Environment and Forestry (DAGRI), University of Florence, 50019 Sesto Fiorentino, Italy; maddalena.fucile@unifi.it (M.F.); giovanbattista.mattii@unifi.it (G.B.M.)

**Keywords:** seaweed extracts, chitosan, humic and fulvic acids, protein hydrolysates, phosphites, plant-growth-promoting rhizobacteria, *Trichoderma* spp.

## Abstract

Climate change and disproportionate anthropogenic interventions, such as the excess of phytopharmaceutical products and continuous soil tillage, are jeopardizing viticulture by subjecting plants to continuous abiotic stress. One of the main physiological repercussions of abiotic stress is represented by the unbalanced redox homeostasis due to the overproduction of reactive oxygen species (ROS), ultimately leading to a state of oxidative stress (detrimental to grape quality). To these are added the direct and indirect damages caused by pathogens (biotic stresses). In light of this scenario, it is inevitable that sustainable techniques and sensitivity approaches for environmental and human health have to be applied in viticulture. Sustainable viticulture can only be made with the aid of sustainable products. Biostimulant (PB) applications (including resistance inducers or elicitors) in the vineyard have become interesting maneuvers for counteracting vine diseases and improving grape quality. These also represent a partial alternative to soil fertilization by improving nutrient absorption and avoiding its leaching into the groundwater. Their role as elicitors has important repercussions in the stimulation of the phenylpropanoid pathway by triggering the activation of several enzymes, such as polyphenol oxidase, lipoxygenase, phenylalanine ammonia-lyase, and peroxidase (with the accumulation of phenolic compounds). The present review paper summarizes the PBs’ implications in viticulture, gathering historical, functional, and applicative information. This work aims to highlight the innumerable beneficial effects on vines brought by these products. It also serves to spur the scientific community to a greater contribution in investigating the response mechanisms of the plant to positive inductions.

## 1. Introduction

Intensive food production for animal and human consumption, for which conventional agricultural systems have been adopted, has led to the haphazard and promiscuous use of agrochemical products, generating several negative and dangerous effects for the agroecosystem, including the conservation of the biodiversity connected to these agricultural systems [1,2,3,4,5]. Farmers commonly use fertilizers to sustain crop yield and profitability [6]. These invalidating impacts involve higher soil contamination, which dwindles its fertility, as well as water pollution [7]; in fact, during the past decades, owing to an excess of nitrogen (N) and phosphorus (P) coming from anthropogenic activities leaching into groundwater or moving into waterways via surface runoff, there was a massive increase in global marine eutrophication [8,9]. Moreover, eutrophication that originates from phytopharmaceutical products (critical values 9–25 μg L^−1^ [10]) leads to the increase in the frequency of anoxic events and the death of several fish species [11]. Contamination with organic pollutants and heavy metals, together with erosion and sustained tillage, diminishes the soil’s quality and signals significant toxicological and environmental threats [12]. In fact, vineyard soils are commonly extremely degraded soils in terms of biochemical properties and are thus more easily affected by contamination [13].

In addition, during the last few decades, some sloping European vineyards were abandoned, which has led to intensive soil erosion and consequent dispersion of pollutants into the environment [14,15]. Increased metal concentration in soils negatively influences the sustainability of agroecosystems [16]. Helling et al. [17] set the critical copper (Cu) concentration, originating from Cu-oxychloride, in soils (above which the population of *Eisenia fetida* earthworm was negatively affected) to 16 mg kg^−1^, a value easily met in several European vineyards. The abuse of fungicides in vineyards is currently a cause of public concern, owing to their resulting presence as residues in water and wine products used for human consumption [18,19]. Cu-based fungicides, such as Cu(OH)_2_, copper oxychloride (3Cu(OH)_2_·CuCl_2_), CuSO_4_, and Cu_2_O, are authorized and necessary for organic grapevine cultivation [20] (according to EC regulation 473/2002, 8 kg Cu ha^−1^, which should be further decreased to 6 kg Cu ha^−1^ after four years of vine cultivation [21]). In addition, synthetic fertilizers and fungicides contain other heavy metals, such as mercury (Hg), cadmium (Cd), arsenic (As), lead (Pb), nickel (Ni), and zinc (Zn), which cause an alarming combination of environmental and health problems [22]. These metals are persistent, toxic, cannot be degraded by microorganisms, and can stockpile through the food chain [23,24]. Some of these metals can migrate to below 100 cm in topsoil [25]. Even though grapes might not be hyperaccumulators of heavy metals [26,27], their uptake and related potential risks still need to be given more attention [28].

Moreover, to the nightmare picture of ground reservoir impoverishment and pollution, the context regarding climate change is annexed [3]. This collective matter about the repercussions of climate change on viticulture is engendered by the well-recognized intense influence that climate has on grapevine ecophysiology and the quality of wine produced [29]. Changes in climate patterns connected to abiotic stresses involve the set of environmental conditions that dwindle growth and yield below optimal standards [30].

One of the main physiological repercussions of abiotic stress is represented by the unbalanced redox homeostasis due to the overproduction of reactive oxygen species (ROS, i.e., leakage of electrons from different cellular compartments), ultimately leading to a state of oxidative stress [31], which modifies the enzymatic activity and the regulation of genes, compromising plant survival. ROS (radicals: superoxide anion (O_2_^−^), peroxyl (RCOO), hydroxyl (OH), and alkoxyl (RO), as well as non-radicals) propagate chain reactions and target biomolecules such as DNA, pigments, lipids, and proteins [32,33]. They can be produced by enzymes such as xanthine oxidase, NADPH-oxidase, peroxidases, and amine oxidase [34]. Furthermore, in grapevines, hydrogen peroxide (H_2_O_2_) is also considered a key regulator of heat shock proteins and many genes of the anthocyanin metabolic pathway [35].

**Figure 1:**
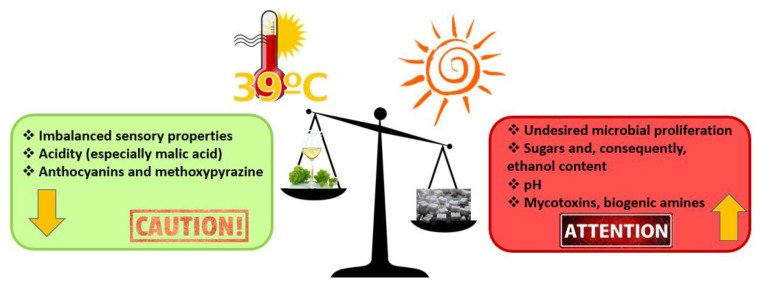
Main effects of rising temperatures on the bunch [40,42,43].

The most common abiotic stresses, which are often interrelated with each other, include high/low temperatures, salinity, drought (water deficit), soil acidification, and excessive radiation exposure [36]. In fact, conventionally, the expression “summer stress” illustrates the combination of several severe abiotic stresses during the summer season, such as high sunlight, water deficit, and high temperature [37]. The conjunction between cluster temperature and sunlight exposure is fundamental in detecting vine metabolism because several biochemical pathways are both temperature- and light-susceptible [38]. Moreover, just think that in the last 10 years, the number of publications on abiotic stresses in *Vitis vinifera* L. increased by about 90%, showing the importance of climate change impacts and abiotic constraints on viticulture, as well as the attempts by researchers towards adapting to these problems [39]. In fact, climate change is exerting a progressively greater influence on grapevine phenology and grape composition (Figure 1), affecting the vinification, microbiology, chemistry, and sensory aspects of wine [40,41].

An upward shift in temperature will dramatically drift the growing season, therewith altering the normal template of grape development with anticipation of blooming, veraison, and harvest. The veraison is a time of particular importance because an excessively early veraison causes shift of the critical ripening period towards the hotter part of the season [42]. The consequences for grape chemistry are considerable, such as an excess of sugar in the berries (and, consequently, alcohol), reduced malic acid concentrations (malolactic fermentation problems), and lower extractable anthocyanin (color and stability problems) and methoxypyrazine levels (lower incidence of herbaceous notes) [44]. In addition, it was shown that sun-exposed clusters were up to 12.4 °C above ambient temperature [45,46], causing damages throughout the growing cycle, such as drying of the berries, sunburn, and reduced yield [47].

Grapevines notice abiotic stress signals and use dynamic and elaborate defense responses, which are either plasticly (irreversible) or elastically (reversible) reliant on the persistence and intensity of the stress (i.e., acute or chronic), as well as the tissue implied [37].

In an anthropized scenario where the winegrower finds, on the one hand, the environmental repercussions due to the excesses of intensive farming and, on the other, climate change, which imposes new challenges, a sustainable and respectful approach towards the vineyard ecosystem becomes necessary in order to obtain healthy and high-quality products [48].

The present paper reviews the different categories of biostimulants and their important implications in viticulture by gathering historical, functional, and applicative information. This work aims to highlight the innumerable beneficial effects on the vine brought by these products. It also serves to propose a greater contribution of scientists to investigating the response mechanisms of the plant to positive inductions.

## 2. History of Biostimulants

The first approach of the “biogenic stimulant” theory started in 1933 in the USSR, and it may be attributed to the Russian doctor V.P. Filatov [49,50,51]. He proposed that, after being stored, biological materials originating from animal or plant organisms accumulate substances that stimulate metabolic processes. When these that were tissues rich in “biogenic stimulators” were introduced into a diseased or stressed organism, the regenerative powers of the treated organism were increased, and the pathological processes were suppressed [49]. During the 1950s, Blagoveshchensky [52,53] defined biogenic stimulants as “organic acids with stimulating effects due to their dibasic properties which can enhance the enzymatic activity in plants”. According to Berlyn and Russo [54], these compounds increased plant growth and vigor by increasing the efficiency of nutrient and water uptake. However, definitions of biostimulants vary greatly, and there are still some arguments surrounding these compounds. Nevertheless, they have been defined as non-fertilized products and hormone-containing substances that can stimulate growth when exogenously applied [55] at low concentrations [56]. A general definition was established by Naumov et al. [57] as a “multi-component balanced system of biologically active substances of metabolic origin on the basis of plant raw materials with a broad spectrum of biological activity”. Herve [58], through his work, constituted the first real modern approach to biostimulants, introducing the concept that the development of new products must be based on characteristics such as being active at low doses, being ecologically benign, and showing reproducible beneficial effects on cultivated plants (“bio-rational products”) [59]. In the late 1990s, Zhang and Schmidt [60] faced the concept of biostimulants as “pre-stress conditioners”, highlighting their effects on photosynthetic efficiency and reduction of spread and intensity of some diseases in higher yields. Using the term minimum quantities (minute quantities) to describe biostimulants, Schmidt et al. [61] intended to distinguish biostimulants from nutrients and soil improvers that also promote growth, but are applied in larger quantities. The action of biostimulants with both hormonal effects (metabolic enhancers) and the protection against abiotic stress induced by antioxidants was explained [62].

The complex multicomponent attitude of biostimulants clearly complicated the discovery of their mechanisms of production, action, registration, and use. However, what is clearly needed is a regulatory mechanism to guarantee that the products are “generally recognized as safe” and are separated from existing categories of products [63]. In fact, in the recent past, the European law rules had completely neglected these products. Only in 2006 did the Italian Legislative Decree 29 April N° 217 “Review of the discipline in fertilizer matter” (G.U n. 141 of the 20 June 2006-Suppl. Ord. N° 152) finally overcome this lack, as it also included “Biostimulants” as “Products that bring to other fertilizer and/or to the soil and/or to the plant, substances that favor or regulate the absorption of the nutrients or correct some physiological anomalies” [64].

Kauffman [65,66,67] introduced a possible taxonomy that included humic substances (HSs), hormone-containing products (HCPs), and amino-acid-containing products (AACPs). Several authors [68,69,70] continued to address the issue in a general way, but it was Basak [71] who pioneered the systematic symposium on biostimulants. In the scientific literature in the following years, the range of substances and modes of action considered was then expanded.

The European Biostimulant Industry Council (EBIC) established a precise definition of biostimulants (June 2011) [72]. At the EU level, biostimulants were defined as “substances or materials (not including nutrients and pesticides) which when applied to the plant, seeds or growth substrate in specific formulations can modify the physiological processes of plants by improving growth, development and/or increase the tolerance to abiotic stresses” [73]. In 2013, the EBIC elaborated a further definition of biostimulants: “Biostimulants are substances and/or microorganisms that applied to the plant or rhizosphere stimulate natural processes that improve the efficiency of absorption and assimilation of nutrients, abiotic stress tolerance, and product quality. Biostimulants have no effect on parasites and pathogens and therefore do not fall under the category of pesticides” [74]. In the same period, Du Jardin [75] gave the first in-depth interpretation of biostimulant science with attention to biostimulant systematization and categorization based on biochemical and physiological function and modes of action. These categorizations and analyses were influential in informing the development of subsequent legislation in the EU. At a regulatory level, amending regulations (EC) no. 1069/2009 and (EC) N.1107/2009 and repealing Regulation (EC) N. 2003/2003, thanks to the introduction of the new European Regulation (EU Reg. 2019/1009), which established rules relating to the availability of fertilizers on the EU market, for the first time, at the legislative level, there was the introduction of the “biostimulants” category, which was previously regulated only by individual countries [76]. According to Du Jardin [75], biostimulants can be classified as follows:

- Humic substances [77]
- Seaweed extracts [78]
- Complex organic materials [79]
- Amino acids and other nitrogenated compounds [80]
- Antitranspirants [81]
- Beneficial chemical elements [82]
- Inorganic salts including phosphorus [83]
- Chitin and derivatives of chitosan [84]

During these years, the study and development of biostimulants were addressed using different modus operandi, such as studies on plant growth and yield [85], non-chemical and chemical composition characterization [86], and application of omics strategies with variations, including microarrays and physiological analyses [87], genomics [88], transcriptomes [89], proteomics [90], and chemical and metabolomics [91].

**Figure 2:**
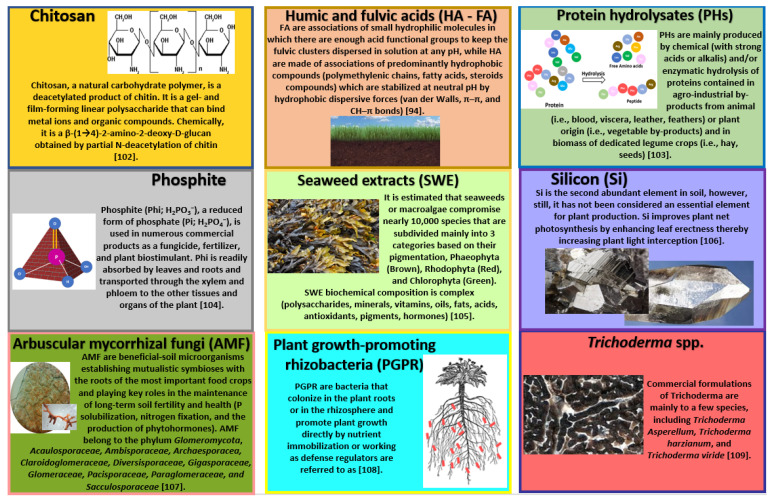
Categories of plant biostimulants [94,102,103,104,105,106,107,108,109].

In 2015, six non-microbial and three microbial categories of plant biostimulants were proposed [92] (Figure 2):

(i) Chitosan [93]
(ii) Humic (HA) and fulvic acids (FAs) [94]
(iii) Protein hydrolysates [95]
(iv) Phosphites [96]
(v) Seaweed extracts [97]
(vi) Silicon [98]
(vii) Arbuscular mycorrhizal fungi (AMF) [99]
(viii) Plant-growth-promoting rhizobacteria (PGPR) [100]
(ix) *Trichoderma* spp. [101].

**Figure 3:**
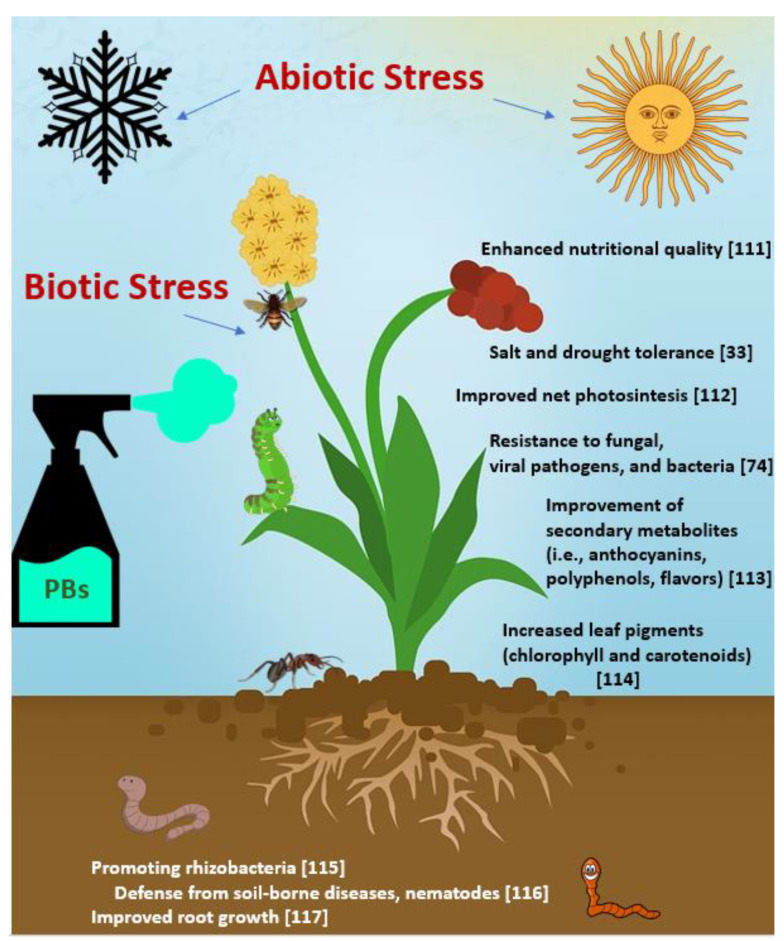
Main effects of biostimulants (PBs) on crops [33,74,111,112,113,114,115,116,117].

Recently, the definition of biostimulants was the following [110]: “A plant biostimulant shall be an EU fertilizing product the function of which is to stimulate plant nutrition processes independently of the product’s nutrient content with the sole aim of improving one or more of the following characteristics of the plant or the plant rhizosphere (Figure 3):

(i) nutrient use efficiency
(ii) tolerance to abiotic stress
(iii) quality traits
(iv) availability of confined nutrients in the soil or rhizosphere.”

However, it is believed that this limited definition could be expanded in light of the countless research described below regarding the resilience of plants to biotic stresses.

## 3. Mechanisms of Action

A biostimulant is described as any microorganism (either beneficial or pathogenic) or substance applied to plants or soil with the aim of increasing nutrient efficiency, abiotic/biotic stress tolerance, and crop quality characteristics [118]. The “mechanism of action” categorizes the biochemical events following application, whereas the “mode of action” distinguishes the main characteristics of a bioactive molecule and the biochemical action that determines its effect in treated plants [119]. Biostimulants (PBs) frequently do not involve a “specific effect on a discrete biochemical or regulatory process”; actually, there are only a small number of PB products for which a definite biochemical target site and known mode of action is recognized [71]. It was suggested that an understanding of the mode of action of PBs on the molecular level needs the receptor-site identification for each regulator and the elucidation of the subsequent reactions [120]. However, this level is often not fulfilled in these products, where these targets cannot be easily achieved [63]. Owing to the heterogeneous nature of the raw materials used for their production, the mechanisms of several PBs remain largely unknown [121].

However, their benefits may be correlated with enzymatic activity changes and antioxidant synthesis. Low concentrations of product enhance the basic biochemical processes in soil and plants, increasing their resistance to several stresses [122]. It was suggested that the active molecules contained in PBs can advance nitrogen (N) assimilation by stimulating Krebs cycle enzymes [123,124]. N_2_-fixing and phosphate-solubilizing bacteria such as *Bacillus* sp. are effectively applied in organic plant cultivation [125]. These bacteria, such as *Azotobacter chroococcum* and *Azospirillum lipoferum*, fix nitrogen and release phytohormones (gibberellic acid and indole acetic acid), which stimulate the absorption of nutrients and net photosynthesis [126].

In addition, PBs’ effects could be ascribed to the movability of plant growth regulators (PGRs) and the power to promote complex regulatory actions that interact among disparate biochemical reactions [127]. Some amino acids could influence growth through their connection to gibberellin biosynthesis [128]; PBs can modify a plant’s hormonal status and employ authority over its growth. For instance, active dry yeast is a natural and safe biofertilizer (a natural cytokinin source) that increases cell division and enlargement, as well as the synthesis of nucleic acid and protein [129]. Peptide signaling is also important during leaf morphogenesis, meristem organization, and defense responses to abiotic or biotic stress. In fact, signaling peptides contained in a plant-derived protein hydrolysate affect meristem organization, callus growth, nodule development, root growth, and leaf-shape regulation [130,131,132].

Environmental stresses, such as heavy metals, drought, and UV radiation, intensify ROS, prompting damage in biomolecule-encompassing proteins. The production of heat-shock proteins (HSPs) is essential for folding and repairing the damaged proteins and promoting cell survival conditions [133]. A protein hydrolysate obtained from alfalfa hydrolysate plants was proved to help *Zea mays* to overcome salinity stress by stimulating enzymes of N metabolism and increasing phenylalanine ammonia-lyase (PAL) activity and flavonoid synthesis [134]. PAL is an important enzyme in the secondary metabolism that changes phenylalanine (C_9_H_11_NO_2_) to trans-cinnamic acid and tyrosine (C_9_H_11_NO_3_) to p-coumaric acid [135]. In plants treated with PBs, the induction of the metabolic phenylpropanoid pathway could represent the reason for why these mixtures can aid plants to overcome stress situations [136]. During the biostimulant activity of alfalfa hydrolysate, the presence of indole-3-acetic acid (IAA) and 1-triacontanol was found. In fact, PBs can motivate the gene expression and activity of several enzymes involved in the tricarboxylic acid cycle (TCA cycle) [137]. In addition, after an oxidative burst response in *Carica papaya* L. [138] and *Ocimum basilicum* L. [139], with hydrogen peroxide (H_2_O_2_) synthesis, the influence of chitosan on the PAL was related to the accumulation of phenolic compounds.

**Figure 4:**
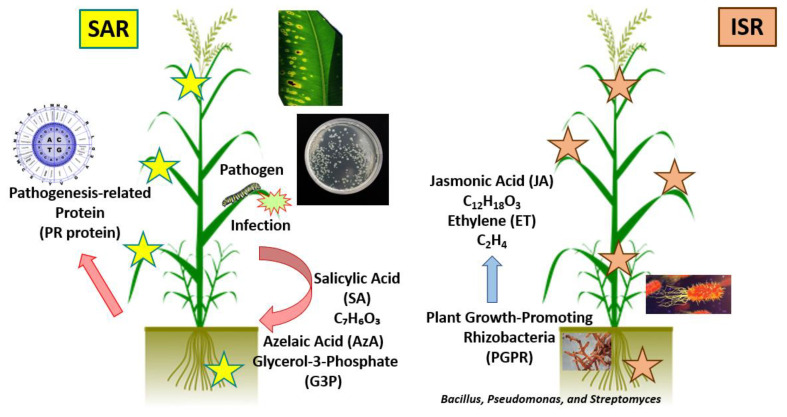
The main plant resistance responses induced at the systemic level. SAR is activated in healthy tissues, distant from the site of infection, and is mediated by SA. ISR is activated in the whole plant following the establishment of symbiosis with beneficial microorganisms at the rhizosphere level and is mediated by JA/ET [141,142,143].

The biosynthesis of phytoalexins (secondary metabolites of low molecular weight with antimicrobial effects) in stressed plants is a subject of study. These compounds are induced by induced systemic resistance (ISR) and systemic acquired resistance (SAR) (Figure 4) [140].

ISR includes a broad metabolic cascade that a plant activates in response to pathogens for protection. The microbe–plant interaction triggers a salicylate-mediated cascade, leading to the long-lasting systemic accumulation of a broad spectrum of defense-related proteins and metabolites, which is called SAR [144].

The application of different biostimulants can trigger the synthesis of these compounds (phytoalexins) through the signal perception for the elicitor signal transduction cascade [145]. Activation is also followed by increases in Ca^2+^ concentration in the cytosol, production of reactive oxygen species (ROS), a localized hypersensitive response (HR), cell wall reinforcement, and stomatal closure [146]. Results suggest that PBs trigger dynamic changes in gene expression and modulate metabolic fluxes in a way that allows plants to perform better.

As regards the penetration of the product into plant tissue, studies on peptide-based biostimulants using radiolabeled amino acids and mathematical models were carried out [147,148]. The components of PBs of animal origin, which were labeled with ^14^C proline and glycine, were demonstrated to penetrate quickly into treated leaves and disseminate to other leaves. Penetration of protein hydrolysates into a plant tissue is energy-dependent [70] and happens by the diffusion of protein through membrane pores [147]. Nevertheless, the precondition for a sufficient penetration is good solubility in water or other suitable solvents. Therefore, surfactants and other additives could be necessary to triumph over solubility and uptake limitations such as active components’ molecular size and lipophilicity [147,148].

However, a wide array of molecular methods is now used to endeavor to distinguish the active compounds found in PBs and probe changes in gene expression, such as metabolomics, microarrays, proteomics and transcriptomic methods.

Nowadays, continuous investments by commercial entities in research and development on PBs, which will serve as a driving force for discoveries in this sector, are considered necessary to lead to the identification of new biological phenomena, pathways, and processes.

## 4. Categories of Biostimulants

This article provides an overview of the topic and focuses on the research of recent years, exclusively analyzing manuscripts on *Vitis vinifera* L.

### 4.1. Seaweed Extracts (SWEs)

The use of seaweeds has a long history originating from Roman and Greek times [149,150]. However, during the last half-century, its cultivation has still developed on an industrial scale following the rapid expansion of production (18M tonnes) and technological developments [151]. Worldwide, SWEs represent more than 33% of the total PB market, and in 2022, the market is vaticinated to reach a value of EUR 894 million [105]. Macroalgae, or seaweeds, include nearly 10,000 species subdivided into three categories based on their pigmentation—*Rhodophyta* (Gray, 1865, Red), *Phaeophyta* (Kjellman, 1891, Brown), and *Chlorophyta* (Reichenbach, 1834, Green) [152]—and are an important source of bioactive peptides, polysaccharides, enzymes, and polyunsaturated fatty acids [153].

Liquid seaweed extracts are produced from seaweed biomass by employing different manufacturing techniques, such as fermentation, acid or alkaline hydrolysis, or cellular disruption under pressure [154]. These methodologies are normally based on soft extractions (low temperature and pressure), with the aim of targeting compounds with low energy consumption, high yield, an optimized extraction process, and reduced waste production [87]. Currently, new technologies, such as supercritical fluid extraction (SFU), ultrasound-assisted extraction (UAE), pressurized liquid extraction (PLE), enzyme-assisted extraction (EAE), and microwave-assisted extraction (MAE), extract biological elements without affecting their efficiency [155]. The seaweed that is most widely employed, which is a fountainhead for PBs, is the brown one, *Ascophyllum nodosum*, a rich sink of bioactive phenolic elements such as phlorotannins and unique polysaccharides (i.e., fucoidans, laminarin, mannitol, and alginic acid) [156]. “*Ascophyllum nodosum* extracts affect the endogenous balance of plant hormones by modulating the hormonal homeostasis, regulate the transcription of a few relevant transporters to alter nutrient uptake and assimilation, stimulate and protect photosynthesis, and dampen stress-induced responses” [157].

SWEs were employed as sustainable tools to improve abiotic stress tolerance, increase grape quality, and enhance the biosynthesis of secondary metabolites in berry skins. Recent shreds of evidence suggest that the beneficial effects of *A. nodosum* treatments on vine acclimation to stressful conditions involve the activation of antioxidant enzymes and secondary metabolic pathways (flavonoid biosynthesis) [158].

The cell walls of seaweeds contain a wide range of polysaccharides, such as β-(1→3)-glucans, which are formed by neutral sugars and acids and can act as elicitors when applied to plant tissues by inducing immunity through the production of reactive oxygen species (ROS), strong enzymatic activity of phenylalanine ammonia-lyase, caffeic acid O-methyltransferase, and lipoxygenase, and the accumulation of salicylic acid and PR proteins [11]. In fact, it was shown that an extract from *Laminaria digitata* (Huds.) J.V.Lamour. applied to grapevine leaves reduced infection by *Botrytis cinerea* Pers.Ex Nocca and Balb. and *Plasmopara viticola* (Berk. and M.A.Curtis) Berl. and De Toni in greenhouse trials by increasing resveratrol and viniferin [159].

In grapevines, foliar application of this brown SWE heightened root development, mineral nutrient uptake (nitrogen status) [160], and vegetative growth expressed by length and leaf area of vine stock [161]. There are also numerous reports on the positive effects of these extracts on yield and grape quality. In fact, in Australia, soil-treated vines (10 L/ha dose from woolly bud to veraison) were improved in wine grape yield by 14.7% [162].

Vines treated with *A. nodosum* extract showed higher flavanol and hydroxycinnamic acid content in both berry skins and leaves, as well as a diminution in the biosynthesis of methoxylated anthocyanins, which are usually accumulated in grapes under environmental limitations [163]. As a consequence of the promotion of the phenylpropanoid metabolism induced by *A. nodosum*, treated vines generated a significantly greater pool of secondary metabolites, including anthocyanin (+0.07 mg/cm^2^) and phenolics (+0.26 mg/cm^2^), on skins [164]. Moreover, it was reported that foliar treatment (rich in oxylipins, phenylalanine, and monosaccharides) in table grapes stimulated the expression of genes involved in anthocyanin biosynthesis [165].

Foliar application of *A. nodosum* extract helped vines’ acclimation to post-veraison water stress by improving their physiological and biochemical performance [166]. Betaines and mannitol in seaweed extracts help plants to survive under stress conditions by improving osmotic adaptability [167]. Under progressive water stress conditions, at a Ψstem value of about −0.65 MPa, foliar treatment positively impacted leaf gas exchange and water-use efficiency (+35% as compared to untreated vines). Photosynthesis was improved (+2.7 μmol CO_2_ m^−2^ s^−1^) via preserved photochemical efficiency (Fv/Fm +0.19) as compared to untreated vines and enhanced leaf anatomical and biochemical traits (+27.3 mg/g DW of leaf soluble sugars and +8% leaf dry matter) [168]. In addition, its action on stomata regulation suggested that this SWE could be a valid tool for restricting leaf damage during extreme temperatures. The treatment increased vines’ transpiration through a reduction of stomatal sensitivity to the vapor-pressure deficit (VPD) (leaf thermoregulation) [169].

The effects of seaweed applications on the volatile composition of white grapes and wines are currently unknown. On cv. Tempranillo Blanco, Gutiérrez-Gamboa et al. [170] showed that a high-dosage (0.50%; *v*/*v*) treatment tended to increase the concentration of (Z)-3-hexen-1-ol, 1-hexanol, and (E)-2-hexen-1-ol in grapes in both seasons, whereas a low-dosage (0.25%; *v*/*v*) application tended to the decrease 2-phenylethanol and 2-phenylethanal content in grapes. In addition, catechin and flavonol (quercetin-3-*O*-glucoside and quercetin-3-*O*-glucuronide) concentrations in berries were increased after the high-dosage application; the treatment affected the trans-caftaric acid, caffeic acid, and total hydroxycinnamic acid content [171]. On the one hand, seaweed treatment at a high dose decreased ρ-cymenene and increased geranyl acetone content in musts. On the contrary, in the following season, high-dose samples presented the highest content of ρ-cymene (85%), nerol oxide (75%), and total terpenoids (36%), whereas low-dose samples presented the lowest content of geraniol (25%) [172]. For these reasons, in order to discern a possible “vintage effect”, it is considered appropriate to make a greater effort to further investigate the effect of this extract on the terpenes of white vines.

In the light of these results, it is considered that SWEs represent a highly efficient and sustainable category of organic non-microbial PBs for improving grapes’ quality and enhancing grapevines’ tolerance to biotic and abiotic stressors, such as extreme temperatures and drought.

### 4.2. Protein Hydrolysates (PHs)

PHs are “mixtures of polypeptides, oligopeptides and amino acids that are manufactured from protein sources using partial hydrolysis” [95]. They are generally originated by chemical (alkaline and acid hydrolysis), enzymatic, and thermal hydrolysis of several animal wastes (i.e., viscera, leather, feathers, blood) and plant biomass (i.e., vegetable by-products). PHs are accessible as liquid extracts or insoluble powders and in granular form and can be applied to roots or as foliar sprays [173].

Chemical hydrolysis is normally selected for creating animal-based PHs by attacking the peptide bonds of proteins and destroying several amino acids, such as tryptophan, cysteine, serine, and threonine. Acid hydrolysis is carried out with hydrochloric and sulphuric acid at >121 °C (high temperatures) and >220.6 kPa pressure. Instead, during alkaline hydrolysis, proteins are solubilized by heating, followed by the addition of calcium, sodium, or potassium hydroxide (alkaline agents). Two critical aspects of chemical hydrolysis are racemization (conversion of free amino acids from the L-form into the D-form) and an increase in the salinity of PHs. Since, in living organisms, the amino acids are only in the L-form, plants cannot directly use D-form amino acids in their metabolism, making PHs potentially toxic for plants [80,95].

Enzymatic hydrolysis is regularly chosen for the generation of plant-based PHs. The result of enzymatic hydrolysis is a mixture of peptides and amino acids with low salinity and a constant composition over time. The hydrolysis is carried out by proteolytic enzymes (e.g., pancreatin, pepsin, papain, ficin, bromelain, alcalase, and flavourzyme) at a low temperature (<60 °C) [95,173,174].

Grapevine production and fruit composition properties were positively influenced by the application of PHs. PHs produced through enzymatic hydrolysis of an organic matrix from lupin (Lup), soybean (Soy), and dairy-mix-based casein (Cas) were tested on *Vitis vinifera* L. cv. Corvina. They were sprayed three times from the fruit set until bunch closure at doses of 1.6–6.4 g L^−1^. The improvement of grapevine performance and cluster weight, including yield, depended on either the PHs’ origin or application dose, with major effects recorded with Lup (1.6 g L^−1^), Soy (6.4 g L^−1^), and Cas (6.4 g L^−1^). As shown by Parrado et al., [175] Cas (1.6 g L^−1^) and Lup (6.4 g L^−1^) showed the ability to increase secondary metabolites synthesized via phenylpropanoid pathways that were involved in plant resistance against stress-condition berry content (total anthocyanin). Soy and Cas significantly decreased the conductance index IG (stress index proportional to stomatal conductance as follows: IG = (T_dry_ − T_canopy_)/(T_canopy_ − T_wet_)), showing the ability of the PHs to reduce stomatal conductance (gs) and transpiration (E), thus ameliorating the tolerance to water stress through the action of abscisic acid (ABA) production, which causes an increase in cytosolic Ca^2+^ concentration [Ca^2+^]_cyt_ [176].

Other results showed that collagen-derived protein thermal hydrolysate applied to roots before imposing water deprivation mitigated the consequences of stress by sustaining vegetative organs’ growth and limiting the extent of cell dehydration [177].

PHs obtained through enzymatic hydrolysis of legume biomass containing 20 g kg^−1^ of urea and 50 g kg^−1^ of nitrogen as peptides and amino acids, as well as 10 g kg^−1^ of soluble potassium (K_2_O), produced deep modifications in leaf metabolomes and proteomes, which maintained higher acidity, thus delaying physiological maturity. PHs significantly modified the concentrations of 69 metabolites compared to those in non-treated vines. Briefly, dehydrospermidine, indole-3-acetyl-phenylalanine, adenine, adenine-ring, (S)-2-amino-3-(3-hydroxy-4-oxo-4h-pyridin-1-yl)-propanoate, 1-iO/i-(4-coumaroyl)-beta-d-glucose, dihydrosterculate, and (5-alpha)-campestan-3-one were upregulated to the system endpoint in comparison with the control vines. At re-watering, PHs significantly upregulated eight metabolites’ concentrations (stigmasterol 3-*O*-β-d-glucoside, few amino-acids, and plastoquinol-9) and downregulated the concentrations of 89 metabolites compared to those in non-treated vines. So, the clearest reaction to the application was a drop in the concentrations of compounds related to the flavanols and their precursors or biosynthetic pathways (downregulation of flavonoids and their precursors in PHs). Moreover, the treatment upregulated the concentration of 3-hidroxy-β-ionone, a metabolite involved in lutein and zeaxanthin cleavage, thus dissipating the excess of energy under summer stress [178].

Soybean and casein hydrolysates induced grapevine immune responses and resistance against *Plasmopara viticola* with the production of resveratrol and its dimer metabolites, δ- and ε-viniferins. They induced a rapid increase in [Ca^2+^]_cyt_ (calcium signaling acts upstream of the MAPK pathway in plant defense responses), elicited marker genes of SA and JA pathways (PR1 and PR6, respectively), and induced the expression of STS (the key gene in resveratrol biosynthesis) [179].

Keeping in mind the negative effects of warming trends that affect traditional wine regions, PHs can be considered valuable tools for improving fruit quality and vineyard sustainability. However, additional work through field trials will be required to further substantiate these results and to convert this knowledge into specific applications that grape growers can unequivocally follow.

### 4.3. Humic Acid (HA) and Fulvic Acid (FA)

Humus is a self-assembled supramolecular association of minute heterogeneous molecules held together by weak hydrophobic linkages [180]. Humic substances (HSs) embody the principal reserve of organic carbon at the Earth’s surface and are formed by biological and chemical transformations of plant and animal matter, as well as microbial metabolism. They handle many important environmental and ecological transactions, such as regulating both soil nitrogen and carbon cycling, plants’ and microorganisms’ growth, and soil structure stabilization. In solution, HSs are a collection of low-molecular-mass components arranging dynamic associations that are stabilized by hydrophobic interactions and hydrogen bonds. HSs, termed hydrophobic acids, can be further operationally divided into two chemical fractions—humic acid (HA) and fulvic acid (FA), depending on the solubility [181] (HA is soluble in aqueous alkaline solutions and precipitates with pH 1–2; in contrast, FA remains in solution after the aqueous alkaline extracts are acidified). A recent definition redesignated FA as associations of little hydrophilic molecules with acid functional groups to hold the fulvic clusters scattered in solution at any pH, whereas HA was redesignated as associations of hydrophobic compounds (fatty acids, polymethylenic chains, steroids compounds) stabilized at neutral pH by hydrophobic dispersive forces (CH–π bonds, van der Waals, and π–π) [182]. By altering the pH and redox potential at the root surface, HSs stimulate root growth and nutrient uptake by promoting secondary transport and overexpression of ion transporters (i.e., cytosolic increase in Ca^2+^ concentration and a regulatory H^+^ efflux activity in the root elongation/differentiation zone) [94].

The environmentally friendly foliar application of HA (derived from vermicompost) was tested at three concentrations: 30, 40, and 50 mL∙L^−1^, the doses of which induced an increase in ATPase synthesis and activity in root cells’ soaring growth, yield, and total soluble solids [183].

Contrary to what Aljabary et al. pointed out [184], organic fertilization with HA (20 mg∙L^−1^) led to an increase in the percentage of phosphorus, nitrogen, and potassium in the petiole leaves of grape seedlings, as well as in the concentration of chlorophyll and the protein percentage in leaves. Adding HA led to an increase in the vine efficiency and its absorption by the roots, thus amplifying macronutrient concentrations in the leaves [185] and anthocyanin content in the juice (mg/100 g fresh weight) [186]. As indirect effects on Superior Seedless grapevines, HA increased soil microbial population, cation exchange capacity, soil structure, tolerance to salinity stress [187], water-holding capacity, aeration, aggregation, permeability, micronutrient transport, and availability [188].

The highest weights in berries and clusters were obtained with HA treatments. These substances also improved the berry detachment and skin rupture forces [189].

The effects of combined foliar application of fulvic acid antitranspirant (FA-AT) were tested [190]. FA-AT controlled the contents of fructose and glucose (mitigating the problems of high alcohol contents) and improved the total phenols and flavonoids in Riesling grapes while it ameliorated the total tannin, individual flavanols, and volatiles in Cabernet Sauvignon grapes (hexyl acetate, linalool) and wine (1-hexanol, 2-phenylethanol, isoamyl alcohol). The weakening of photosynthesis explained the reduction in individual anthocyanins in the grapes (downregulation of gene expression of phenylalanine-aminolyase) in Cabernet Sauvignon.

The combined action of FA with microelements (Mg + K or Fe SO_4_. 7H_2_O at 0.36 g + Zn SO_4_.7H_2_O at 0.18 g + MnSO_4_. H_2_O at 0.18 g) resulted in a significant increase in different parameters, such as in budburst, fertility, vegetative growth, shoot length, leaf surface area, total chlorophyll content, yield/vine, total sugars, and total anthocyanin content in berry skin, while it gave the lowest decrease in acidity [191,192].

Thanks to the induction of resistance to *Botrytis cinerea* through the activation of the phenylpropanoid pathway, FA can be used as an activator of plant defense responses to control postharvest gray mold in table grapes. In fact, FA generated a higher accumulation of phenolic compounds and the activities of cinnamate-4-hydroxylase (C4H), phenylalanine ammonia-lyase (PAL), and 4-coumarate-CoA ligase (4CL) with upregulation of genes related to phenylpropanoid biosynthesis (4CL, STS, PAL, C4H, ROMT, and CHS) [193].

HA and HF could be used to improve the soil organic matter since these PBs play an important role in increasing soil fertility and sustainability. These findings could provide a practical basis for evaluating precision viticulture applications to enhance grapevine development, yield, and berry quality under abiotic stress where cold damage and degraded soil conditions commonly restrict the viticulture.

### 4.4. Chitosan

Chitosan is the deacetylated form of chitin (a co-polymer of N-acetyl-d-glucosamine and d-glucosamine). It is a natural biopolymer present in insect exoskeletons, fungal cell walls, and crustacean shells. Chitosan promotes several defensive genes in plants (e.g., pathogenesis-related genes, such as glucanase and chitinase). In addition, it induces several enzymes in the reactive oxygen species scavenging system (catalase, superoxide dismutase, and peroxidase). Chitosan was used as a PB to stimulate plant growth, abiotic stress tolerance, and pathogen resistance [93].

Chitosan’s effect in inducing vine defense mechanisms can be associated with its ability to widen the intracellular content of a large spectrum of antioxidants (e.g., resveratrol) mainly by strictly regulating the proteomic expression profile. In chitosan-treated samples, 73 proteins consistently changed. In particular, de-novo synthesis and/or accumulation of stilbene synthase proteins were promoted by chitosan, which also stimulated endogenous accumulation of trans-resveratrol. Chitosan treatment strongly increased the expression of 11 proteins of the pathogenesis-related protein-10 family, as well as their mRNA levels [194].

In the Thompson Seedless variety, Clotrimazole-loaded chitosan nanoparticles reached a significant drug entrapment efficiency of 94.7%, revealing a promising antifungal effect against *Candida albicans* and *Aspergillus niger* with average inhibition zone diameters of 74 and 72 mm. The product can be used as a novel anti-dermatophytic agent with an elevated wound-healing capacity [195]. In addition, it induced the bio-control efficacy of *Pichia anomala* by enhancing the activities of disease-defense-related enzymes, such as chitinase and ascorbate peroxidase, and decreasing the formation of hydrogen peroxide and malondialdehyde (responsible for the deterioration of fruits) [196].

Chitosan treatment in berries altered the regulation of reactive oxygen species with up-accumulation of Cu/Zn superoxide dismutase and glyoxal oxidase, hence promoting defense and lignification processes in a hypersensitive response. Furthermore, enzymes involved in anthocyanin, rather than stilbene phytoalexins, accumulated in treated clusters. By eliciting defense mechanisms, there was an increase in stilbenes, hydroperoxide lyase, oxylipins, pentacyclic triterpenoids ursolate, oleanoate, and betulinate [197].

The application of chitosan led to increased levels of polyphenols, anthocyanins, and tannins in Tinto Cão berries, as well as polyphenols and tannins in Touriga Franca berries, thus increasing the antioxidant potential of the berries. In chitosan-treated berries, the following ROS pathway genes were found to be upregulated: amine oxidase (AO), iron-superoxide dismutase (Fe-SOD), catalase (CAT), glutathione reductase (GR), glutaredoxin (Grx), respiratory burst oxidase (Rboh), copper-zinc-superoxide dismutase (Cu/Zn-SOD), peroxidase (POD), and polyphenol oxidase (PPO). So, it was shown that chitosan induced the synthesis of phenolic compounds and also acted as a facilitator for transfer of polyphenols from the leaves to the berries [198].

The impact of pre-harvest foliar spraying with chitosan (2.0% and 3.0%) resulted in a reduction of the decay index by forming a semi-permeable barrier on the surface of the fruit, postponing maturity and senescence, and decreasing the activity of cell-wall-degrading enzymes (pectin methylesterase and polygalacturonase enzymes). In addition, the results showed anthocyanin accumulation, which was associated with increased sugar accumulation, and an increase in malondialdehyde, polyphenol oxidase, firmness, antioxidant capacity, peroxidase, and vitamin C [199].

To understand the effect of chitosan on the levels of phenolic compounds in the berry skin of red grapes (cv. Tinto Cão) during veraison, grapevines were treated with chitosan (0.01% in 0.01% acetic acid). The results showed that monomeric anthocyanins, catechin, rutin, and quercetin-3-O-galactoside significantly increased in berry skins after treatment with chitosan. In addition, in leaves and berry skins, chitosan treatment upregulated several target genes (i.e., PAL, UFGT, ABCC1, CHS, F3H, ANR, GST, and MATE1) that encode key enzymes and transporters involved in secondary metabolic pathways [200]. On Mouhtaro cv. (a Greek red indigenous variety), chitosan treatment increased the abundance of the beneficial lactic acid bacteria (*Lactobacillus* genus) and improved the polyphenolic picture [201].

Chitosan raised the total acetal (1,1-diethoxyethane) and alcohol levels, thus improving the volatile profile, flavor, and taste of Groppello wine (chemical fungicide residual levels may alter yeast metabolism and the biosynthesis of volatile compounds). The reduction of aldehydes by enhanced alcohol dehydrogenase activity and the increase in elicitors stimulating glycosidases increased alcohols and odorant compounds, respectively [202]. These results were not confirmed in other work. The application of foliar-chitosan elicitor decreased the synthesis of positive grape volatile compounds (C13 norisoprenoids, benzenoids, and esters) [203].

Chitosan plays a key role as an elicitor against pathogen infestation, giving a sustainable alternative to chemical pesticides by improving the synthesis of secondary metabolites. However, its effect on grape volatile compounds has been little investigated. Furthermore, based on the findings, chitosan treatments could be considered as suitable preferences for extending the marketable interval of table grapes and downsizing post-harvest deprivations.

### 4.5. Trichoderma spp.

*Trichoderma* spp. helps against environmental stresses, such as drought and salinity, by reinforcing plant growth and reprogramming gene expression in roots and shoots, thus improving nutrient and water acquisition. It was also used as a beneficial microorganism due to its capacity to inhibit many fungal plant pathogens [101]. These rhizosphere microorganisms function by producing large quantities of extracellular enzymes (i.e., 6-pentyl-2H-pyran-2-one and auxin indole-3-acetic acid) that lead to the death of negative plant pathogenic fungi and reduce chemical inputs, thus promoting conservation of natural resources [204].

By behaving as endophytes, *Trichoderma* strains showed their potential as biological control agents by reducing the colonization of *Phaeoacremonium minimum* (Tul. and C.Tul.) Gramaje, L.Mostert, and Crous [205,206]. Furthermore, the microscopic observation of histochemistry revealed an increased accumulation of callose, lignin, and hydrogen peroxide and an upregulation of the activities of defense enzymes, such as peroxidase, phenylalanine ammonia-lyase, and 1,3-glucanase, highlighting the protection induced by *Trichoderma harzianum* Rifai in response to *Plasmopara viticola* [207]. *Trichoderma* Fleming (1822), which was positively used for biological control of *Erysiphe necator* Schw. (*Uncinula necator* (Schw.) Burr.), tested positive for the production of ammonia, hydrogen cyanide, indole acetic acid, siderophore, phosphate, chitinase, β-1,3-glucanase, cellulase, amylase, and protease (plant-growth-promoting bio-chemicals) [208]. Among the symptoms of esca complex, *Trichoderma asperellum* Samuels, Lieckf., and Nirenberg and *Trichoderma gamsii* Samuels and Druzhin. were demonstrated to downsize the impact of light tiger-stripe symptoms and apoplexy, but no differences were found in the medium-to-severe symptoms [209]. In addition, in another study, it was shown that *Trichoderma asperellum*, *T. harzianum*, and *T. atroviride* Bissett reduced downy mildew (*P. viticola*) severity on grapevine leaf disks by producing volatile organic compounds (VOCs), such as α-farnesene, 2-pentylfuran, cadinene, 1,3-octadiene, 6-pentyl-2H-pyran-2-one, 6-pentyl-2H-pyran-2-one, and 2-pentylfuran [210].

In addition, *Trichoderma* acts directly as an entomopathogen through parasitism and the production of insecticidal secondary metabolites, such as repellent metabolites and antifeedant compounds. It was demonstrated to produce secondary metabolites of a volatile nature, such as 6-pentyl-α-pyrone, which caused 100% mortality in *Tetranychus urticae* Koch (1836) in 48 h (the red spider mite) [211]. Soil *T. harzianum* and *T. gamsii* applications were considered as control agents against *Xylotrechus arvicola* (Olivier, 1795) in vineyards to inhibit egg development, prevent larvae from boring into vines, and kill adults [212].

*Trichoderma* strains used in biological control products usually exhibit high efficiency in the control of plant diseases (biotic stress). However, nowadays, since many of the studies are carried out in vitro, their behavior under field conditions is difficult to predict.

### 4.6. Plant-Growth-Promoting Rhizobacteria (PGPR)

These beneficial microorganisms, which enhance the resistance to biotic and abiotic stress factors in plants, are prevalent near plant roots in an area called the rhizosphere and include the following genera: *Alcaligenes*, *Mesorhizobium*, *Rhizobium*, *Rhodococcus*, *Azospirillum*, *Azotobacter*, *Agrobacterium*, *Bacillus*, *Bradyrhizobium*, *Burkholderia*, *Caulobacter*, *Chromobacterium*, *Enterobacter*, *Herbaspirillum*, *Klebsiella*, *Micrococcus*, *Pseudomonas*, *Arthrobacter*, *Erwinia*, *Flavobacterium*, and *Serratia*. PGPR can be divided into symbiotic bacteria (living within plant tissues and exchanging metabolites) and free-living rhizobacteria (living outside of plant tissues and promoting plant growth) based on their interactions with plants [213]. Owing to their beneficial effects on plant health by suppressing phytopathogens and accelerating nutrient assimilation, among the bacteria investigated for biocontrol, increased attention was given to actinobacteria. These filamentous bacteria produce a broad range of bioactive compounds that act as plant-growth-promoting substances (siderophores, antifungal compounds, hydrolytic enzymes, hydrocyanic acid, and ammonia gas) that are antagonists of the hosted pathogens, and they synthesize phytohormones, fix atmospheric nitrogen, solubilize inorganic phosphate, and inhibit stress-induced ethylene by producing the enzyme 1-aminocyclopropane-1-carboxylic acid (ACC) deaminase [214,215]. In addition, they can indirectly intensify plant growth by weakening the deleterious effects of phytopathogens by engendering systemic resistance (ISR) and production of antimicrobial compounds (i.e., fengycin, chitinase, bacteriocin, zwittermicin, and cell-wall-degrading enzymes) [216].

Grapevine trunk diseases (GTDs) are unfortunately a serious threat to the sustainability of vineyards. Esca complex (*Phaeomoniella chlamydospora*, *Phaeoacremonium minimum*, and *Fomitoporia mediterranea*), Eutypa dieback (*Eutypa lata,* but also *Eutypa* sp. and *Eutypella* sp.), and Botryosphaeria dieback (*Botryosphaeria dothidea*, *Diplodia seriata*, and *Neofusicoccum parvum*) are the three main GTDs [217].

Screening based on antagonistic and plant-growth-promotion abilities of certain strains—Streptomyces, Saccharothrix, Nocardia, Nocardiopsis, Actinoplanes, Lentzea, Promicromonospora, Nonomuraea, Saccharopolyspora, and Streptosporangium—showed an appreciable antagonistic activity against both Paeomoniella chlamydospora and Phaeoacremonium minimum. These strains were able to produce siderophores, ammonia, indole acetic acid, ACC deaminase, cellulase, and amylase, as well as to fix N_2_ [218].

A new chemical control strategy for GTDs is to develop site-targeted fungicides to protect grapevine vascular tissues in combined use with biological agents. The effects of a phloem-mobile derivative of the fungicide Fenpiclonil with plant-growth-promoting rhizobacteria (*Paraburkholderia phytofirmans* in the *Neofusicoccum parvum* strain Bourgogne) were evaluated. The combined treatment (systemic fenpiclonil derivative + *Paraburkholderia phytofirmans*) evidenced a strong activation of host immune responses, especially for defense-related genes and phenylpropanoid pathways, giving the highest control efficiency against the GTD pathogen (*N. parvum* strain Bourgogne) [219].

A recent study also confirmed the beneficial effects of this interaction. Grapevines were inoculated with the plant-growth-promoting rhizobacterium *Ensifer meliloti* TSA4. *E. meliloti* inoculation increased the growth parameters of the vine plants, improved phosphorus absorption, and facilitated P uptake from the soil, suggesting a successful PGPR–plant association [220].

During a standard process of grapevine nursery propagation, epiphytic and rhizospheric inoculation by the PGPR strain *Pseudomonas protegens* MP12 was effective in controlling an artificially induced *Botrytis cinerea* infection in detached leaves. The success of rhizospheric and leaf colonization in vine plants suggests a potential for the future exploitation of *P. protegens* MP12 as a biofertilizer and biopesticide [221].

However, it is believed that further investigations are needed to improve our understanding of the mechanisms of interaction between the PGPRs that occur in plants and, in particular, with systemic fungicides. We suggest a greater commitment by researchers in multi-year field trials and in different viticultural areas to appreciate any benefits, including those for bunches by, for example, adopting epiphytic inoculations in the pre-harvest period to prevent infections by *Botrytis cinerea* or *Plasmopara viticola* in the berries. The effectiveness of this possible treatment could reduce or eliminate the use of synthetic organic fungicides (e.g., dithiocarbamates).

### 4.7. Arbuscular Mycorrhizal Fungi (AMF)

AMF are an important group of soil microorganisms that can establish symbiotic interrelationships with vine roots, and they represent an integral component of the vineyard ecosystem with applications for sustainable viticulture. Vineyard-living microbiota and mutualistic plant–microbe interactions affect the biological quality of soils, adaptation of grapevines to changing environments, and the response to abiotic stresses by determining wine quality [222].

A recent study showed that the AMF from grapevine vineyard root samples were dominated by *Glomus* sp., followed by *Claroideoglomus* sp. [223].

Leaves of grapevine plants inoculated with *Funneliformis mosseae* showed an increase in volatile organic compounds (VOCs) related to plant defense under pathogen attack or linked to water stress, such as geraniol, (E)–2–hexenal, 3–hexenal, benzaldehyde, and methyl salicylate. On the contrary, C13–norisoprenoids decreased strongly in mycorrhizal vines [224].

Grapevines in different soil conditions showed a positive response to AMF inoculation, which alleviated the toxic effects of metals and increased photosynthesis and plant growth [225]. In particular, the *Glomeraceae* family can moderate high concentrations of copper in soil [226].

Inoculation of vineyard soil with AMF can be a convenient strategy for reestablishing land mycorrhizal potential, helping vines to better withstand heatwaves [227], and improving water-use efficiency [228].

A particular interaction was discussed by Landi et al. [229]. Their study suggested a relationship between esca disease and native AMF in grapevine roots. The AMF colonization intensity showed a higher value in esca-symptomatic vines (from 24.6% to 61.3%) than in neighboring asymptomatic vines (from 17.4% to 57.6%).

Although the interest in arbuscular mycorrhizal fungal associations has increased in recent years as the demands for sustainable cropping systems have become more pressing, these associations with specific crops (especially vineyards) have received little attention, and nowadays, knowledge is limited.

### 4.8. Silicon (Si)

Silicon constitutes a notable portion of soil as silicate or aluminum silicate, but most cannot be directly absorbed by plants despite its abundance. At concentrations between 0.1 and 2.0 mM (pH < 9), H_4_SiO_4_ (silicic acid) is willingly absorbed into the root system. Its concentration in plants’ aboveground parts ranges between 0.1% and 10.0% dry weight [230]. It is regarded as a beneficial element that increases plant resistance against various abiotic and biotic stresses [231].

Si boosts plant vigor by improving root mass and density. It improves plant cell wall strength, structural integrity, and drought and frost resistance, and it strengthens plants’ natural pest- and disease-fighting systems [232].

Soil application of colloidal silicon (544 kg Si/ha) increased plant-available Si, while the foliar application (428 kg Si/ha) augmented the total silicon concentrations in leaves, yield, and cluster weight (Grüner Veltliner cv.) [233].

Another study positively tested the application of calcite–silicon-mediated particle film (3% *v*/*v*) at veraison as a reliever for a drought-induced increase in leaf temperature, thus contributing to improved leaf functionality, yield, and grape composition traits [234].

Foliar application of Si (1000 mg/L) increased the potassium percentage in leaves, antioxidant enzyme activities, yield per vine, percentage of soluble solids, total anthocyanin, and total phenols, while it reduced the percentage of total acidity. In addition, Si reduced downy mildew disease severity as compared with that in untreated control vines by acting as a physical barrier in cell walls and preventing the penetration of fungal hyphae into host tissues. Concerning the photosynthetic pigments, they were increased in grapevines sprayed with silicon (chlorophyll a (0.815 mg/g FW), chlorophyll b (616 mg/g FW), total chlorophyll (1.431 mg/g FW), and total carotenoid (0.103 mg/g FW)) [235].

Furthermore, a recent experimental design that included three irrigation water regimes (40, 70, 100% of drip irrigation water requirement), as well as chitosan + silicon applications (125 mg L^−1^ Si, 250 mg L^−1^ Chi, and 125 mg L^−1^ Si+250 mg L^−1^ Chi), showed that the Chi + Si treatment under severe drought had an ameliorative effect on cell ultrastructure compared with drought-affected plants (well-developed chloroplasts and increased plastoglobules) [236].

Moreover, Si was reported to reduce the effects of freezing on vines (foliar and soil applications). This may be attributed to the enhancement of non-photochemical quenching and more protection of PSII from photodamage following the foliar spray. In addition, Si application significantly decreased the membrane damage because of efficient scavenging by peroxidase [237]. Under salt stress, the addition of Si also improved all growth parameters and increased the pigments and photosynthetic rates by increasing the maximum yield and potential photochemical efficiency of the photochemical reactions in photosystem II [238].

Therefore, it is believed that the integration of silicon is an excellent corroborant for mitigating any dysfunctions in photosynthesis caused by abiotic or biotic stress.

### 4.9. Phosphite

Phosphite (H_2_PO_3_^−^; Phi) and its conjugate, phosphorous acid (H_3_PO_3_), have more progressively been adopted as supplemental biostimulants, fertilizers, and pesticides. As a PB, Phi improves nutrient uptake and assimilation, abiotic stress tolerance, and grape quality [96]. Several studies showed the efficiency of Phi in controlling plant diseases caused by oomycetes (i.e., *Plasmopara viticola*, *Phytophthora*, and *Pythium*), bacteria (i.e., *Streptomyces scabies*, *Erwinia carotovora*, and *E. amylovora*), and fungi (*Fusarium*, *Armillaria mellea*, *Phakopsora euvitis*, and *Elsinoe ampelina*) [104,239,240,241,242,243].

In a recent study, the effects on molecular-defense-related genes and polyphenol content (stilbenes and flavanols) were revealed. Phi tended to modulate the defense responses. In fact, in response to a downy mildew inoculation, the pre-treated leaves overproduced pterostilbene, piceids, and ε-viniferin. The elicitor triggered the overexpression of two PR protein genes: VvPR5 (thaumatin-like protein) and VvPR4 (chitinase). In addition, Phi induced the genes VvPR5 and VvPR6 (serine protease inhibitor). The treatment led to the overexpression of several genes that are directly involved in the biosynthesis of callose (VvCAL) and the modification of the cell wall with pectin methylesterase (VvPECT) and cinnamoyl-CoA reductase (VvCAD) (genes involved in cell wall reinforcement) [244].

Since the reduction of antimicrobial treatments and the application of environmentally friendly treatments, such as Phi, are impelling challenges, to undertake more sustainable agriculture, a constant and assiduous commitment on the part of research is needed to disseminate more and more results in this area.

## 5. Conclusions

Meeting one’s own needs without harming the needs of future generations is a cardinal principle of society and is also the basis of the current new convention of viticulture: conservativeness and sustainability. Preserving viticultural ecosystems, conserving water resources, enhancing soil elements, assisting plants against abiotic and biotic stresses, and preventing erosive events and contamination by pesticides are fundamental agricultural concepts and practices for ensuring the healthiness of the products and avoiding irreversible damage. With the aid of these substances, environmental health, vines, and humans are more protected by minimizing costs in terms of agricultural inputs. The use of seaweed extracts, humic substances, chitosan, exudates, and other extracts preserves, defends, and strengthens vines without harming the ecology or human health. A sustainable approach will boost the growth of grapes’ marketability, giving a higher value to the product (for example, with an organic label). In fact, these findings provide evidence for the potential of at least partially replacing conventional fungicides, rendering viticulture more sustainable in terms of soil protection and biodiversity. The improvement of the soil elements thanks to the help of phosphite, the lower exposure to pesticides mitigated by *Trichoderma* and plant-growth-promoting rhizobacteria, and the better resistance to drought and high temperatures promoted by seaweed extracts represent sustainable approaches to preserving the viticulture ecosystem from irreversible consequences. However, some limitations in the research are highlighted, such as the scarcity of multi-year field tests in different viticultural areas (PGPR), the clarification of the relationship between esca disease and arbuscular mycorrhizal fungi, and the scarcity of studies concerning grape quality due to anthocyanin fractionation and flavor detection (AMF, Si, Phi) Finally, this review would like to put an emphasis on spurring the scientific community to a greater contribution to the investigation of the response mechanisms of plants to positive inductions.

## References

[1] Neset T.S., Wiréhn L., Klein N., Käyhkö J., Juhola S. (2019). Maladaptation in Nordic agriculture. Clim. Risk Manag..

[2] Gutiérrez-Gamboa G., Verdugo-Vásquez N., Díaz-Gálvez I. (2019). Influence of type of management and climatic conditions on productive behavior, oenological potential, and soil characteristics of a ‘Cabernet Sauvignon’ vineyard. Agronomy.

[3] Gutiérrez-Gamboa G., Zheng W., Martínez de Toda F. (2021). Strategies in vineyard establishment to face global warming in viticulture: A mini review. J. Sci. Food Agric..

[4] Perrino E.V., Ladisa G., Calabrese G. (2014). Flora and plant genetic resources of ancient olive groves of Apulia (southern Italy). Genet. Resour. Crop Evol..

[5] Perrino E.V., Calabrese G. (2018). Vascular flora of vineyards in the DOC area “Gioia del Colle” (Apulia, Southern Italy): Preliminary data. Nat. Croat..

[6] Della Chiesa S., Genova G., la Cecilia D., Niedrist G. (2019). Phytoavailable phosphorus (P_2_O_5_) and potassium (K_2_O) in topsoil for apple orchards and vineyards, South Tyrol, Italy. J. Maps.

[7] Parris K. (2011). Impact of agriculture on water pollution in OECD countries: Recent trends and future prospects. Int. J. Water Resour. Dev..

[8] Howarth R.W. (2008). Coastal nitrogen pollution: A review of sources and trends globally and regionally. Harmful Algae.

[9] Ngatia L., Grace J.M., Moriasi D., Taylor R. (2019). Nitrogen and phosphorus eutrophication in marine ecosystems. Monit. Mar. Pollut..

[10] Ramos M.C., Martínez-Casasnovas J.A. (2006). Nutrient losses by runoff in vineyards of the Mediterranean Alt Penedès region (NE Spain). Agric. Ecosyst. Environ..

[11] Gutiérrez-Gamboa G., Moreno-Simunovic Y. (2021). Seaweeds in viticulture: A review focused on grape quality. Ciênc Técnic Vitiviníc.

[12] Komárek M., Čadková E., Chrastný V., Bordas F., Bollinger J.C. (2010). Contamination of vineyard soils with fungicides: A review of environmental and toxicological aspects. Environ. Int..

[13] Miguéns T., Leirós M.C., Gil-Sotres F., Trasar-Cepeda C. (2007). Biochemical properties of vineyard soils in Galicia, Spain. Sci. Total Environ..

[14] Nóvoa-Muñoz J.C., Queijeiro J.M.G., Blanco-Ward D., Álvarez-Olleros C., Martínez-Cortizas A., García-Rodeja E. (2007). Total copper content and its distribution in acid vineyards soils developed from granitic rocks. Sci. Total Environ..

[15] Fernández-Calviño D., Rodríguez-Suárez J.A., López-Periago E., Arias-Estévez M., Simal-Gándara J. (2008). Copper content of soils and river sediments in a winegrowing area, and its distribution among soil or sediment components. Geoderma.

[16] Munishi L.K., Ndakidemi P.A., Blake W., Comber S., Hutchinson T.H. (2021). Toxic metals in East African agroecosystems: Key risks for sustainable food production. J. Environ..

[17] Helling B., Reinecke S.A., Reinecke A.J. (2000). Effects of the fungicide copper oxychloride on the growth and reproduction of *Eisenia fetida* (Oligochaeta). Ecotoxicol. Environ. Saf..

[18] Jacobson A.R., Dousset S., Guichard N., Baveye P., Andreux F. (2005). Diuron mobility through vineyard soils contaminated with copper. Environ. Pollut..

[19] Gachene C.K., Nyawade S.O., Karanja N.N. (2020). Soil and water conservation: An overview. Zero Hunger.

[20] Hummes A.P., Bortoluzzi E.C., Tonini V., da Silva L.P., Petry C. (2019). Transfer of copper and zinc from soil to grapevine-derived products in young and centenarian vineyards. Water Air Soil Pollut..

[21] European Commission (2002). EC (European Commission) Regulation 473/2002 Amending Annexes I, II and VI to Council Regulation (EEC) No 2092/91 on Organic Production of Agricultural Products and Indications Referring Thereto on Agricultural Products and Foodstuffs and Laying Down Detailed Rules as Regards the Transmission of Information on the Use of Copper Compounds.

[22] Alengebawy A., Abdelkhalek S.T., Qureshi S.R., Wang M.Q. (2021). Heavy metals and pesticides toxicity in agricultural soil and plants: Ecological risks and human health implications. Toxics.

[23] Zang F., Wang S., Nan Z., Ma J., Zhang Q., Chen Y., Li Y. (2017). Accumulation, spatio-temporal distribution, and risk assessment of heavy metals in the soil-corn system around a polymetallic mining area from the Loess Plateau, northwest China. Geoderma.

[24] Wang Y., Li F., Song J., Xiao R., Luo L., Yang Z., Chai L. (2018). Stabilization of Cd-, Pb-, Cu-and Zn-contaminated calcareous agricultural soil using red mud: A field experiment. Environ. Geochem. Health.

[25] Li P., Lin C., Cheng H., Duan X., Lei K. (2015). Contamination and health risks of soil heavy metals around a lead/zinc smelter in southwestern China. Ecotoxicol. Environ. Saf..

[26] Milićević T., Urošević M.A., Relić D., Vuković G., Škrivanj S., Popović A. (2018). Bioavailability of potentially toxic elements in soil–grapevine (leaf, skin, pulp and seed) system and environmental and health risk assessment. Sci. Total Environ..

[27] Milićević T., Urošević M.A., Relić D., Jovanović G., Nikolić D., Vergel K., Popović A. (2021). Environmental pollution influence to soil-plant–air system in organic vineyard: Bioavailability, environmental, and health risk assessment. Environ. Sci. Pollut. Res..

[28] Yang L., Ren Q., Zheng K., Jiao Z., Ruan X., Wang Y. (2022). Migration of heavy metals in the soil-grape system and potential health risk assessment. Sci. Total Environ..

[29] Venios X., Korkas E., Nisiotou A., Banilas G. (2020). Grapevine responses to heat stress and global warming. Plants.

[30] Skirycz A., Inzé D. (2010). More from less: Plant growth under limited water. Curr. Opin. Biotechnol..

[31] Sharma P., Jha A.B., Dubey R.S., Pessarakli M. (2012). Reactive oxygen species, oxidative damage, and antioxidative defense mechanism in plants under stressful conditions. J. Bot..

[32] Møller I.M., Jensen P.E., Hansson A. (2007). Oxidative modifications to cellular components in plants. Annu Rev. Plant Biol..

[33] Sharma H.S., Fleming C., Selby C., Rao J.R., Martin T. (2014). Plant biostimulants: A review on the processing of macroalgae and use of extracts for crop management to reduce abiotic and biotic stresses. J. Appl. Phycol..

[34] Schmidt R., Schippers J.H. (2015). ROS-mediated redox signaling during cell differentiation in plants. Biochim. Biophys. Acta (BBA) Gen. Subj..

[35] Guo M., Liu J.H., Ma X., Luo D.X., Gong Z.H., Lu M.H. (2016). The plant heat stress transcription factors (HSFs): Structure, regulation, and function in response to abiotic stresses. Front. Plant Sci..

[36] Tester M., Bacic A. (2005). Abiotic stress tolerance in grasses. From model plants to crop plants. Plant Physiol..

[37] Cramer G.R., Urano K., Delrot S., Pezzotti M., Shinozaki K. (2011). Effects of abiotic stress on plants: A systems biology perspective. BMC Plant Biol..

[38] Spayd S.E., Tarara J.M., Mee D.L., Ferguson J.C. (2002). Separation of sunlight and temperature effects on the composition of *Vitis vinifera* cv. Merlot berries. Am. J. Enol. Vitic..

[39] Bernardo S., Dinis L.T., Machado N., Moutinho-Pereira J. (2018). Grapevine abiotic stress assessment and search for sustainable adaptation strategies in Mediterranean-like climates. A review. Agron. Sustain. Dev..

[40] De Orduna R.M. (2010). Climate change associated effects on grape and wine quality and production. Food Res. Int..

[41] Ferrandino A., Pagliarani C., Carlomagno A., Novello V., Schubert A., Agati G. (2017). Improved fluorescence-based evaluation of flavonoid in red and white winegrape cultivars. Aust. J. Grape Wine Res..

[42] Keller M. (2010). Managing grapevines to optimise fruit development in a challenging environment: A climate change primer for viticulturists. Aust. J. Grape Wine Res..

[43] Berbegal C., Fragasso M., Russo P., Bimbo F., Grieco F., Spano G., Capozzi V. (2019). Climate changes and food quality: The potential of microbial activities as mitigating strategies in the wine sector. Fermentation.

[44] Mozell M.R., Thach L. (2014). The impact of climate change on the global wine industry: Challenges & solutions. Wine Econ. Policy.

[45] Millar A.A. (1972). Thermal regime of grapevines. Am. J. Enol. Vitic..

[46] Smart R.E., Sinclair T.R. (1976). Solar heating of grape berries and other spherical fruits. Agric. Meteorol..

[47] Wahid A., Gelani S., Ashraf M., Foolad M.R. (2007). Heat tolerance in plants: An overview. Environ. Exp. Bot..

[48] Cataldo E., Salvi L., Sbraci S., Storchi P., Mattii G.B. (2020). Sustainable viticulture: Effects of soil management in *Vitis vinifera*. Agronomy.

[49] Filatov V.P. (1944). Tissue Therapy in Ophthalmology. Am. Rev. Sov. Med..

[50] Gordon D.M. (1974). The treatment of retinitis pigmentosa with special reference to the Filatov method. Am. J. Ophthalmol..

[51] Filatov V.P. (1951). Tissue treatment. (Doctrine on biogenic stimulators). II. Hypothesis of tissue therapy, or the doctrine on biogenic stimulators. Priroda.

[52] Blagoveshchensky A.V. (1955). Biogenic stimulants in agriculture. Priroda.

[53] Blagoveshchensky A.V. (1956). Biogenic stimulants and biochemical nature of their action. Bull. Main Bot. Gard..

[54] Berlyn G.P., Russo R.O. (1990). The use of organic biostimulants to promote root growth. Belowground Ecol..

[55] Schmidt R.E. (1992). Biostimulants. Grounds Maint..

[56] Goatley J.M., Schmidt R.E. (1991). Biostimulator enhancement of Kentucky bluegrass sod. HortScience.

[57] Naumov G.F., Bozhkov A.I., Leontovich V.P., Sklyar A.I., Belous A.M. (1993). Polyfunctionality of allelopathic substance allelostim. Dokl. Akad. Nauk Ukr..

[58] Herve J.J. (1994). Biostimulants, a new concept for the future; prospects offered by the chemistry of synthesis and biotechnology. C. R. Acad. Agric. Fr..

[59] Elliott M.L., Prevatte M. (1996). Response of ‘Tifdwarf’ Bermudagrass to Seaweed-derived Biostimulants. HortTechnology.

[60] Zhang X., Schmidt R. (1999). Biostimulating turfgrasses. Grounds Maint..

[61] Schmidt R.E., Ervin E.H., Zhang X. (2003). Questions and answers about biostimulants. Golf Course Manag..

[62] Doak S.O., Schmidt R.E., Ervin E.H. (2005). Metabolic enhancer impact on creeping bentgrass leaf sodium and physiology under salinity. Int. Turfgrass Soc. Res. J..

[63] Yakhin O.I., Lubyanov A.A., Yakhin I.A., Brown P.H. (2017). Biostimulants in plant science: A global perspective. Front. Plant Sci..

[64] Ciavatta C., Cavani L. (2006). Problematiche per l’inserimento dei biostimolanti nella legislazione dei fertilizzanti. Fertil. Agrorum.

[65] Kauffman G.L., Kneivel D.P., Watschke T.L. (2007). Effects of a biostimulant on the heat tolerance associated with photosynthetic capacity, membrane thermostability, and polyphenol production of perennial ryegrass. Crop Sci..

[66] Crouch I.J., Smith M.T., Van Staden J., Lewis M.J., Hoad G.V. (1992). Identification of auxins in a commercial seaweed concentrate. J. Plant Physiol..

[67] Zhang X., Ervin E.H. (2004). Cytokinin-containing seaweed and humic acid extracts associated with creeping bentgrass leaf cytokinins and drought resistance. Crop Sci..

[68] Apone F., Arciello S., Colucci G., Filippini L., Portoso D. (2006). Alle radici della biostimolazione: Indagini scientifiche a supporto. Fertil. Agrorum.

[69] Kumar D., Shivay Y.S. (2008). Definitional Glossary of Agricultural Terms.

[70] Parrado J., Bautista J., Romero E.J., García-Martínez A.M., Friaza V., Tejada M. (2008). Production of a carob enzymatic extract: Potential use as a biofertilizer. Bioresour. Technol..

[71] Basak A. (2008). Biostimulators–Definitions, Classification and Legislation. Biostimulators in Modern Agriculture: General Aspects.

[72] Toscano S., Romano D., Massa D., Bulgari R., Franzoni G., Ferrante A. (2018). Biostimulant applications in low input horticultural cultivation systems. Italus Hortus.

[73] Du Jardin P. (2012). The Science of Plant Biostimulants—A Bibliographic Analysis.

[74] Drobek M., Frąc M., Cybulska J. (2019). Plant biostimulants: Importance of the quality and yield of horticultural crops and the improvement of plant tolerance to abiotic stress—A review. Agronomy.

[75] Du Jardin P. (2015). Plant biostimulants: Definition, concept, main categories and regulation. Sci. Hortic..

[76] EU Regulation (2019). Regulation (EU) 2019/1009 of the European Parliament and of the Council of 5 June 2019 laying down rules on the making available on the market of EU fertilising products and amending Regulations (EC) No 1069/2009 and (EC) No 1107/2009 and repealing Regulation (EC) No 2003/2003. Off. J. Eur. Union.

[77] MacCarthy P. (2001). The principles of humic substances. Soil Sci..

[78] Khan W., Rayirath U.P., Subramanian S., Jithesh M.N., Rayorath P., Hodges D.M., Critchley A.T., Craigie J.S., Norrie J., Prithiviraj B. (2009). Seaweed extracts as biostimulants of plant growth and development. J. Plant Growth Regul..

[79] Nardi S., Pizzeghello D., Schiavon M., Ertani A. (2016). Plant biostimulants: Physiological responses induced by protein hydrolyzed-based products and humic substances in plant metabolism. Sci. Agric..

[80] Popko M., Michalak I., Wilk R., Gramza M., Chojnacka K., Górecki H. (2018). Effect of the new plant growth biostimulants based on amino acids on yield and grain quality of winter wheat. Molecules.

[81] Mphande W., Kettlewell P.S., Grove I.G., Farrell A.D. (2020). The potential of antitranspirants in drought management of arable crops: A review. Agric. Water Manag..

[82] Calvo P., Nelson L., Kloepper J.W. (2014). Agricultural uses of plant biostimulants. Plant Soil.

[83] Aeron A., Dubey R.C., Maheshwari D.K. (2021). Next-Generation biofertilizers and novel biostimulants: Documentation and validation of mechanism of endophytic plant growth-promoting rhizobacteria in tomato. Arch. Microbiol..

[84] Shahrajabian M.H., Chaski C., Polyzos N., Tzortzakis N., Petropoulos S.A. (2021). Sustainable Agriculture Systems in Vegetable Production Using Chitin and Chitosan as Plant Biostimulants. Biomolecules.

[85] Tian S., Lu L., Xie R., Zhang M., Jernstedt J., Hou D., Ramsier C., Brown P. (2015). Supplemental macronutrients and microbial fermentation products improve the uptake and transport of foliar applied zinc in sunflower (*Helianthus annuus* L.) plants. Studies utilizing micro X-ray florescence. Front. Plant Sci..

[86] Aremu A.O., Stirk W.A., Kulkarni M.G., Tarkowská D., Turečková V., Gruz J., Šubrtová M., Pěnčík A., Novák O., Doležal K. (2015). Evidence of phytohormones and phenolic acids variability in garden-waste-derived vermicompost leachate, a well-known plant growth stimulant. Plant Growth Regul..

[87] Jannin L., Arkoun M., Etienne P., Laîné P., Goux D., Garnica M., Fuentes M., San Francisco S., Baigorri R., Cruz F. (2013). Brassica napus growth is promoted by *Ascophyllum nodosum* (L.) Le Jol. seaweed extract: Microarray analysis and physiological characterization of N, C, and S metabolisms. J. Plant Growth Regul..

[88] Santaniello A., Giorgi F.M., Di Tommaso D., Di Tommaso G., Piaggesi A., Perata P. (2013). Genomic approaches to unveil the physiological pathways activated in Arabidopsis treated with plant-derived raw extracts. Acta Hortic..

[89] Goñi O., Fort A., Quille P., Mckeown P.C., Spillane C., O’Connell S. (2016). Comparative transcriptome analysis of two Ascophyllum nodosum extract biostimulants: Same seaweed but different. J. Agric. Food Chem..

[90] Martínez-Esteso M.J., Vilella-Antón M.T., Sellés-Marchart S., Martínez-Márquez A., Botta-Català A., Piñol-Dastis R., Bru-Martínez R. (2016). A DIGE proteomic analysis of wheat flag leaf treated with TERRA-SORB® foliar, a free amino acid high content biostimulant. J. Integr. Omics.

[91] Ertani A., Pizzeghello D., Francioso O., Sambo P., Sanchez-Cortes S., Nardi S. (2014). Capsicum chinensis L. growth and nutraceutical properties are enhanced by biostimulants in a long-term period: Chemical and metabolomic approaches. Front. Plant Sci..

[92] Colla G., Rouphael Y. (2015). Biostimulants in horticulture. Sci. Hortic..

[93] Pichyangkura R., Chadchawan S. (2015). Biostimulant activity of chitosan in horticulture. Sci. Hortic..

[94] Canellas L.P., Olivares F.L., Aguiar N.O., Jones D.L., Nebbioso A., Mazzei P., Piccolo A. (2015). Humic and fulvic acids as biostimulants in horticulture. Sci. Hortic..

[95] Colla G., Nardi S., Cardarelli M., Ertani A., Lucini L., Canaguier R., Rouphael Y. (2015). Protein hydrolysates as biostimulants in horticulture. Sci. Hortic..

[96] Gómez-Merino F.C., Trejo-Téllez L.I. (2015). Biostimulant activity of phosphite in horticulture. Sci. Hortic..

[97] Battacharyya D., Babgohari M.Z., Rathor P., Prithiviraj B. (2015). Seaweed extracts as biostimulants in horticulture. Sci. Hortic..

[98] Savvas D., Ntatsi G. (2015). Biostimulant activity of silicon in horticulture. Sci. Hortic..

[99] Rouphael Y., Franken P., Schneider C., Schwarz D., Giovannetti M., Agnolucci M., De Pascale S., Bonini P., Colla G. (2015). Arbuscular mycorrhizal fungi act as biostimulants in horticultural crops. Sci. Hortic..

[100] Ruzzi M., Aroca R. (2015). Plant growth-promoting rhizobacteria act as biostimulants in horticulture. Sci. Hortic..

[101] López-Bucio J., Pelagio-Flores R., Herrera-Estrella A. (2015). *Trichoderma* as biostimulant: Exploiting the multilevel properties of a plant beneficial fungus. Sci. Hortic..

[102] Islam N., Dmour I., Taha M.O. (2019). Degradability of chitosan micro/nanoparticles for pulmonary drug delivery. Heliyon.

[103] Maini P. (2006). The experience of the first biostimulant, based on amino acids and peptides: A short retrospective review on the laboratory researches and the practical results. Fertil. Agrorum.

[104] Wu L., Gao X., Xia F., Joshi J., Borza T., Wang-Pruski G. (2019). Biostimulant and fungicidal effects of phosphite assessed by GC-TOF-MS analysis of potato leaf metabolome. Physiol. Mol. Plant Pathol..

[105] EL Boukhari M.E., Barakate M., Bouhia Y., Lyamlouli K. (2020). Trends in seaweed extract based biostimulants: Manufacturing process and beneficial effect on soil-plant systems. Plants.

[106] Azad M.O.K., Park B.S., Adnan M., Germ M., Kreft I., Woo S.H., Park C.H. (2021). Silicon biostimulant enhances the growth characteristics and fortifies the bioactive compounds in common and Tartary buckwheat plant. J. Crop Sci. Biotechnol..

[107] Giovannini L., Palla M., Agnolucci M., Avio L., Sbrana C., Turrini A., Giovannetti M. (2020). Arbuscular mycorrhizal fungi and associated microbiota as plant biostimulants: Research strategies for the selection of the best performing inocula. Agronomy.

[108] Kumari B., Mallick M.A., Solanki M.K., Solanki A.C., Hora A., Guo W. (2019). Plant growth promoting rhizobacteria (PGPR): Modern prospects for sustainable agriculture. Plant Health under Biotic Stress.

[109] Fernando D., Milagrosa S., Francisco C., Francisco M. (2018). Biostimulant activity of *Trichoderma saturnisporum* in melon (*Cucumis melo*). HortScience.

[110] Rouphael Y., Colla G. (2020). Biostimulants in agriculture. Front. Plant Sci..

[111] Van Oosten M.J., Pepe O., De Pascale S., Silletti S., Maggio A. (2017). The role of biostimulants and bioeffectors as alleviators of abiotic stress in crop plants. Chem. Biol. Technol. Agric..

[112] Desoky E.S.M., ElSayed A.I., Merwad A.R.M., Rady M.M. (2019). Stimulating antioxidant defenses, antioxidant gene expression, and salt tolerance in Pisum sativum seedling by pretreatment using licorice root extract (LRE) as an organic biostimulant. Plant Physiol. Biochem..

[113] Gutiérrez-Gamboa G., Romanazzi G., Garde-Cerdán T., Pérez-Álvarez E.P. (2019). A review of the use of biostimulants in the vineyard for improved grape and wine quality: Effects on prevention of grapevine diseases. J. Sci. Food Agric..

[114] Bulgari R., Cocetta G., Trivellini A., Vernieri P., Ferrante A. (2015). Biostimulants and crop responses: A review. Biol. Agric. Hortic..

[115] Backer R., Rokem J.S., Ilangumaran G., Lamont J., Praslickova D., Ricci E., Subramanian S., Smith D.L. (2018). Plant growth-promoting rhizobacteria: Context, mechanisms of action, and roadmap to commercialization of biostimulants for sustainable agriculture. Front. Plant Sci..

[116] D’Addabbo T., Laquale S., Perniola M., Candido V. (2019). Biostimulants for plant growth promotion and sustainable management of phytoparasitic nematodes in vegetable crops. Agronomy.

[117] Vernieri P., Borghesi E., Ferrante A., Magnani G. (2005). Application of biostimulants in floating system for improving rocket quality. J. Food Agric. Environ..

[118] Ganugi P., Martinelli E., Lucini L. (2021). Microbial biostimulants as a sustainable approach to improve the functional quality in plant-based foods: A review. Curr. Opin. Food Sci..

[119] Aliferis K.A., Jabaji S. (2011). Metabolomics—A robust bioanalytical approach for the discovery of the modes-of-action of pesticides: A review. Pestic. Biochem. Physiol..

[120] Halmann M. (1990). Synthetic plant growth regulators. Adv. Agron..

[121] Rathore S.S., Chaudhary D.R., Boricha G.N., Ghosh A., Bhatt B.P., Zodape S.T., Patolia J.S. (2009). Effect of seaweed extract on the growth, yield and nutrient uptake of soybean (Glycine max) under rainfed conditions. S. Afr. J. Bot..

[122] Rafiee H., Naghdi Badi H., Mehrafarin A., Qaderi A., Zarinpanjeh N., Sękara A., Zand E. (2016). Application of plant biostimulants as new approach to improve the biological responses of medicinal plants-A critical review. Med. Plant Res..

[123] Schiavon M., Ertani A., Nardi S. (2008). Effects of an alfalfa protein hydrolysate on the gene expression and activity of enzymes of the tricarboxylic acid (TCA) cycle and nitrogen metabolism in *Zea mays* L.. J. Agric. Food Chem..

[124] Ertani A., Cavani L., Pizzeghello D., Brandellero E., Altissimo A., Ciavatta C., Nardi S. (2009). Biostimulant activity of two protein hydrolyzates in the growth and nitrogen metabolism of maize seedlings. J. Plant. Nutr. Soil Sci..

[125] Lugtenberg B., Kamilova F. (2009). Plant-growth-promoting rhizobacteria. Annu. Rev. Microbiol..

[126] Mahfouz S.A., Sharaf-Eldin M.A. (2007). Effect of mineral vs. biofertilizer on growth, yield, and essential oil content of fennel [*Foeniculum vulgare* Mill.]. Int. Agrophys..

[127] Machado V.P.D.O., Pacheco A.C., Carvalho M.E.A. (2014). Effect of biostimulant application on production and flavonoid content of marigold (*Calendula officinalis* L.). Rev. Ceres.

[128] Van Overbeek J. (1966). Plant Hormones and Regulators: Gibberellins, cytokinins, and auxins may regulate plant growth via nucleic acid and enzyme synthesis. Science.

[129] Mady M.A. (2009). Effect of foliar application with yeast extract and zinc on fruit setting and yield of faba bean (*Vicia faba* L.). J. Biol. Chem. Environ. Sci..

[130] Colla G., Svecova E., Rouphael Y., Cardarelli M., Reynaud H., Canaguier R., Planques B. (2013). Effectiveness of a plant-derived protein Hydrolysate to improve crop performances under different growing conditions. Acta Hortic..

[131] Paul K., Sorrentino M., Lucini L., Rouphael Y., Cardarelli M., Bonini P., Moreno M.B.M., Reynaud H., Canaguier R., Trtílek M. (2019). A combined phenotypic and metabolomic approach for elucidating the biostimulant action of a plant-derived protein hydrolysate on tomato grown under limited water availability. Front. Plant Sci..

[132] Ceccarelli A.V., Miras-Moreno B., Buffagni V., Senizza B., Pii Y., Cardarelli M., Rouphael Y., Colla G., Lucini L. (2021). Foliar application of different vegetal-derived protein hydrolysates distinctively modulates tomato root development and metabolism. Plants.

[133] Calabrese V., Giordano J., Ruggieri M., Berritta D., Trovato A., Ontario M.L., Bianchini R., Calabrese E.J. (2016). Hormesis, cellular stress response, and redox homeostasis in autism spectrum disorders. J. Neurosci. Res..

[134] Ertani A., Schiavon M., Altissimo A., Franceschi C., Nardi S. (2011). Phenol-Containing organic substances stimulate phenylpropanoid metabolism in *Zea mays*. J. Plant. Nutr. Soil Sci..

[135] Nishiyama Y., Yun C.S., Matsuda F., Sasaki T., Saito K., Tozawa Y. (2010). Expression of bacterial tyrosine ammonia-lyase creates a novel p-coumaric acid pathway in the biosynthesis of phenylpropanoids in Arabidopsis. Planta.

[136] Xu L., Trinh H.K., Geelen D. (2020). Biostimulant mode of action: Impact of PBs on molecular level. The Chemical Biology of Plant Biostimulants.

[137] Ertani A., Sambo P., Nicoletto C., Santagata S., Schiavon M., Nardi S. (2015). The use of organic biostimulants in hot pepper plants to help low input sustainable agriculture. Chem. Biol. Technol. Agric..

[138] Ali A., Mohamed M.T.M., Siddiqui Y. (2012). Control of anthracnose by chitosan through stimulation of defence-related enzymes in Eksotika II papaya (*Carica papaya* L.) fruit. J. Biol. Life Sci..

[139] Kim H.J., Chen F., Wang X., Rajapakse N.C. (2005). Effect of chitosan on the biological properties of sweet basil (*Ocimum basilicum* L.). J. Agric. Food Chem..

[140] Pretali L., Bernardo L., Butterfield T.S., Trevisan M., Lucini L. (2016). Botanical and biological pesticides elicit a similar induced systemic response in tomato (*Solanum lycopersicum*) secondary metabolism. Phytochemistry.

[141] Pieterse C.M., Leon-Reyes A., Van der Ent S., Van Wees S.C. (2009). Networking by small-molecule hormones in plant immunity. Nat. Chem. Biol..

[142] Lim G.H., Shine M.B., de Lorenzo L., Yu K., Cui W., Navarre D., Hunt A.G., Lee J.Y., Kachroo A., Kachroo P. (2016). Plasmodesmata localizing proteins regulate transport and signaling during systemic acquired immunity in plants. Cell Host Microbe.

[143] Luo J., Xia W., Cao P., Xiao Z.A., Zhang Y., Liu M., Zhan C., Wang N. (2019). Integrated transcriptome analysis reveals plant hormones jasmonic acid and salicylic acid coordinate growth and defense responses upon fungal infection in poplar. Biomolecules.

[144] Kamle M., Borah R., Bora H., Jaiswal A.K., Singh R.K., Kumar P. (2020). Systemic Acquired Resistance (SAR) and Induced Systemic Resistance (ISR): Role and mechanism of action against phytopathogens. Fungal Biotechnology and Bioengineering.

[145] Vargas-Hernandez M., Macias-Bobadilla I., Guevara-Gonzalez R.G., Romero-Gomez S.D.J., Rico-Garcia E., Ocampo-Velazquez R.V., Alvarez-Arquieta L.L., Torres-Pacheco I. (2017). Plant hormesis management with biostimulants of biotic origin in agriculture. Front. Plant Sci..

[146] Han X., Xi Y., Zhang Z., Mohammadi M.A., Joshi J., Borza T., Wang-Pruski G. (2021). Effects of phosphite as a plant biostimulant on metabolism and stress response for better plant performance in *Solanum tuberosum*. Ecotoxicol. Environ. Saf..

[147] Kolomazník K., Pecha J., Friebrová V., Janáčová D., Vašek V. (2012). Diffusion of biostimulators into plant tissues. Heat Mass Transf..

[148] Pecha J., Fürst T., Kolomazník K., Friebrová V., Svoboda P. (2012). Protein biostimulant foliar uptake modeling: The impact of climatic conditions. AIChE J..

[149] Rindi F., Soler-Vila A., Guiry M.D. (2012). Taxonomy of marine macroalgae used as sources of bioactive compounds. Marine Bioactive Compounds.

[150] Arioli T., Mattner S.W., Winberg P.C. (2015). Applications of seaweed extracts in Australian agriculture: Past, present and future. J. Appl. Phycol..

[151] Winberg P.C., Fitton H.J., Stringer D., Karpiniec S.S., Gardiner V.A. (2014). Controlling seaweed biology, physiology and metabolic traits in production for commercially relevant bioactives in glycobiology. Adv. Bot. Res..

[152] Cornish M.L., Monagail M.M., Critchley A.T. (2020). The Animal Kingdom, Agriculture and Seaweeds. J. Mar. Sci. Eng..

[153] Okolie C.L., Mason B., Critchley A.T. (2018). Seaweeds as a source of proteins for use in pharmaceuticals and high-value applications. Novel Proteins for Food, Pharmaceuticals, and Agriculture: Sources, Applications and Advances.

[154] Offei F., Mensah M., Thygesen A., Kemausuor F. (2018). Seaweed bioethanol production: A process selection review on hydrolysis and fermentation. Fermentation.

[155] Michalak I., Chojnacka K. (2015). Algae as production systems of bioactive compounds. Eng. Life Sci..

[156] Bajpai S., Shukla P.S., Asiedu S., Pruski K., Prithiviraj B. (2019). A biostimulant preparation of brown seaweed *Ascophyllum nodosum* suppresses powdery mildew of strawberry. Plant Pathol. J..

[157] De Saeger J., Van Praet S., Vereecke D., Park J., Jacques S., Han T., Depuydt S. (2020). Toward the molecular understanding of the action mechanism of *Ascophyllum nodosum* extracts on plants. J. Appl. Phycol..

[158] Cai Z., Kastell A., Mewis I., Knorr D., Smetanska I. (2012). Polysaccharide elicitors enhance anthocyanin and phenolic acid accumulation in cell suspension cultures of Vitis vinifera. Plant Cell Tissue Organ Cult. (PCTOC).

[159] Aziz A., Poinssot B., Daire X., Adrian M., Bézier A., Lambert B., Joubert J.M., Pugin A. (2003). Laminarin elicits defense responses in grapevine and induces protection against *Botrytis cinerea* and *Plasmopara viticola*. Mol. Plant Microbe Interact..

[160] Taskos D., Stamatiadis S., Yvin J.C., Jamois F. (2019). Effects of an *Ascophyllum nodosum* (L.) Le Jol. extract on grapevine yield and berry composition of a Merlot vineyard. Sci. Hortic..

[161] Popescu G.C., Popescu M. (2014). Effect of the brown alga Ascophyllum nodosum as biofertilizer on vegetative growth in grapevine (*Vitis vinifera* L.). Curr. Trends Nat. Sci..

[162] Arioli T., Mattner S.W., Hepworth G., McClintock D., McClinock R. (2021). Effect of seaweed extract application on wine grape yield in Australia. J. Appl. Phycol..

[163] Salvi L., Brunetti C., Cataldo E., Niccolai A., Centritto M., Ferrini F., Mattii G.B. (2019). Effects of Ascophyllum nodosum extract on *Vitis vinifera*: Consequences on plant physiology, grape quality and secondary metabolism. Plant Physiol. Biochem..

[164] Frioni T., Sabbatini P., Tombesi S., Norrie J., Poni S., Gatti M., Palliotti A. (2018). Effects of a biostimulant derived from the brown seaweed Ascophyllum nodosum on ripening dynamics and fruit quality of grapevines. Sci. Hortic..

[165] Petoumenou D.G., Patris V.E. (2021). Effects of Several Preharvest Canopy Applications on Yield and Quality of Table Grapes (*Vitis vinifera* L.) Cv. Crimson Seedless. Plants.

[166] Salvi L., Brunetti C., Cataldo E., Storchi P., Mattii G.B. (2020). Eco-physiological traits and phenylpropanoid profiling on potted *Vitis vinifera* L. cv Pinot noir subjected to *Ascophyllum nodosum* treatments under post-veraison low water availability. Appl. Sci..

[167] Abbas M., Anwar J., Zafar-ul-Hye M., Iqbal Khan R., Saleem M., Rahi A.A., Danish S., Datta R. (2020). Effect of seaweed extract on productivity and quality attributes of four onion cultivars. Horticulturae.

[168] Frioni T., VanderWeide J., Palliotti A., Tombesi S., Poni S., Sabbatini P. (2021). Foliar vs. soil application of *Ascophyllum nodosum* extracts to improve grapevine water stress tolerance. Sci. Hortic..

[169] Tombesi S., Frioni T., Sabbatini P., Poni S., Palliotti A. (2021). *Ascophyllum nodosum* extract improves leaf thermoregulation by reducing stomatal sensitivity to VPD in *Vitis vinifera* L.. J. Appl. Phycol..

[170] Gutiérrez-Gamboa G., Garde-Cerdán T., Rubio-Bretón P., Pérez-Álvarez E.P. (2020). Seaweed foliar applications at two dosages to Tempranillo blanco (*Vitis vinifera* L.) grapevines in two seasons: Effects on grape and wine volatile composition. Food Res. Int..

[171] Gutiérrez-Gamboa G., Garde-Cerdán T., Martínez-Lapuente L., Costa B.S.D., Rubio-Bretón P., Pérez-Álvarez E.P. (2020). Phenolic composition of Tempranillo Blanco (*Vitis vinifera* L.) grapes and wines after biostimulation via a foliar seaweed application. J. Sci. Food Agric..

[172] Gutiérrez-Gamboa G., Garde-Cerdán T., Rubio-Bretón P., Pérez-Álvarez E.P. (2021). Effects on must and wine volatile composition after biostimulation with a brown alga to Tempranillo grapevines in two seasons. J. Sci. Food Agric..

[173] Colla G., Hoagland L., Ruzzi M., Cardarelli M., Bonini P., Canaguier R., Rouphael Y. (2017). Biostimulant action of protein hydrolysates: Unraveling their effects on plant physiology and microbiome. Front. Plant Sci..

[174] Moreno-Hernández J.M., Benítez-García I., Mazorra-Manzano M.A., Ramírez-Suárez J.C., Sánchez E. (2020). Strategies for production, characterization and application of protein-based biostimulants in agriculture: A review. Chil. J. Agric. Res..

[175] Parrado J., Escudero-Gilete M.L., Friaza V., García-Martínez A., González-Miret M.L., Bautista J.D., Heredia F.J. (2007). Enzymatic vegetable extract with bio-active components: Influence of fertiliser on the colour and anthocyanins of red grapes. J. Sci. Food Agric..

[176] Boselli M., Bahouaoui M.A., Lachhab N., Sanzani S.M., Ferrara G., Ippolito A. (2019). Protein hydrolysates effects on grapevine (*Vitis vinifera* L., cv. Corvina) performance and water stress tolerance. Sci. Hortic..

[177] Meggio F., Trevisan S., Manoli A., Ruperti B., Quaggiotti S. (2020). Systematic Investigation of the Effects of a Novel Protein Hydrolysate on the Growth, Physiological Parameters, Fruit Development and Yield of Grapevine (*Vitis vinifera* L., cv Sauvignon Blanc) under Water Stress Conditions. Agronomy.

[178] Bavaresco L., Lucini L., Squeri C., Zamboni M., Frioni T. (2020). Protein hydrolysates modulate leaf proteome and metabolome in water-stressed grapevines. Sci. Hortic..

[179] Lachhab N., Sanzani S.M., Adrian M., Chiltz A., Balacey S., Boselli M., Ippolito A., Poinssot B. (2014). Soybean and casein hydrolysates induce grapevine immune responses and resistance against *Plasmopara viticola*. Front. Plant Sci..

[180] Nebbioso A., Piccolo A. (2012). Advances in humeomics: Enhanced structural identification of humic molecules after size fractionation of a soil humic acid. Anal. Chim. Acta.

[181] Derrien M., Lee Y.K., Park J.E., Li P., Chen M., Lee S.H., Lee S.H., Lee J.B., Hur J. (2017). Spectroscopic and molecular characterization of humic substances (HS) from soils and sediments in a watershed: Comparative study of HS chemical fractions and the origins. Environ. Sci. Pollut. Res..

[182] Islam M.A., Morton D.W., Johnson B.B., Angove M.J. (2020). Adsorption of humic and fulvic acids onto a range of adsorbents in aqueous systems, and their effect on the adsorption of other species: A review. Sep. Purif. Technol..

[183] Popescu G.C., Popescu M. (2018). Yield, berry quality and physiological response of grapevine to foliar humic acid application. Bragantia.

[184] Aljabary A.M.O., Al-Baytie M.R.S., Ahmed Z.S. (2018). Effect of number eyes left after pruning, fertilization with humic acid and spraying with gibberellic acid in some mineral content of vineyards thompson cv. vitis viniferal. Plant Arch..

[185] Imam N.M.A.A., Al-Obaidi H.S.F. (2020). Effect of adding the chemical fertilizer NPK and humic acid on the growth and mineral percentage for seedlings of three grape cultivars (*Vitis vinifera* L.). Euphrates J. Agric. Sci..

[186] Al-Atrushy S.M., Mustafa S.A. (2016). Foliar Application of Humic Acid, Iron and Sprays Number on Chemical Quality of Grape (*Vitis vinifera* L.) cv. *TAIFI*. ICNS.

[187] EL Ghayaty S.H., Abdrabboh G.A., Hamdy A.E., Ahmed A.F. (2019). Effect of soil applications anti-salinity agent on growth, yield and fruit quality of superior seedless grapevines (*Vitis vinifera* L.). Al-Azhar J. Agric. Res..

[188] Asgharzade A., Babaeian M. (2012). Investigating the effects of humic acid and acetic acid foliar application on yield and leaves nutrient content of grape (*Vitis vinifera*). Afr. J. Microbiol. Res..

[189] Sabir A., Sagdıç K., Sabır F.K. (2021). Vermicompost, humic acid and urea pulverizations as sustainable practices on the face of climatic extremities to increase grape yield and quality. Int. J. Agric. Nat. Sci..

[190] Li W., Yao H., Chen K., Ju Y., Min Z., Sun X., Cheng Z., Liao Z., Zhang K., Fang Y. (2021). Effect of foliar application of fulvic acid antitranspirant on sugar accumulation, phenolic profiles and aroma qualities of Cabernet Sauvignon and Riesling grapes and wines. Food Chem..

[191] El-Boray M.S., Mostafa M.F., Shaltout A.D., Hassan K.H. (2015). Influence of fulvic acid plus some microelements and microorganisms on yield and quality characteristics of superior seedless grapevines. J. Plant Prod..

[192] Mostafa M.F.M., EL-Boray M.S., El-Baz E.L., Omar A.S. (2017). Effect of Fulvic Acid and Some Nutrient Elements on King Ruby Grapevines Growth, Yield and Chemical Properties of Berries. J. Plant Prod..

[193] Xu D., Deng Y., Xi P., Yu G., Wang Q., Zeng Q., Jiang Z., Gao L. (2019). Fulvic acid-induced disease resistance to *Botrytis cinerea* in table grapes may be mediated by regulating phenylpropanoid metabolism. Food Chem..

[194] Ferri M., Tassoni A., Franceschetti M., Righetti L., Naldrett M.J., Bagni N. (2009). Chitosan treatment induces changes of protein expression profile and stilbene distribution in *Vitis vinifera* cell suspensions. Proteomics.

[195] Elshaer E.E., Elwakil B.H., Eskandrani A., Elshewemi S.S., Olama Z.A. (2021). Novel Clotrimazole and *Vitis vinifera* loaded chitosan nanoparticles: Antifungal and Wound Healing Efficiencies. Saudi J. Biol. Sci..

[196] Godana E.A., Yang Q., Wang K., Zhang H., Zhang X., Zhao L., Abdelhai M.H., Legrand N.N.G. (2020). Bio-Control activity of Pichia anomala supplemented with chitosan against Penicillium expansum in postharvest grapes and its possible inhibition mechanism. LWT.

[197] Lucini L., Baccolo G., Rouphael Y., Colla G., Bavaresco L., Trevisan M. (2018). Chitosan treatment elicited defence mechanisms, pentacyclic triterpenoids and stilbene accumulation in grape (*Vitis vinifera* L.) bunches. Phytochemistry.

[198] Singh R.K., Soares B., Goufo P., Castro I., Cosme F., Pinto-Sintra A.L., Ines A., Oliveira A.A., Falco V. (2019). Chitosan upregulates the genes of the ROS pathway and enhances the antioxidant potential of grape (*Vitis vinifera* L. ‘Touriga Franca’ and ’Tinto Cão’) tissues. Antioxidants.

[199] Nia A.E., Taghipour S., Siahmansour S. (2021). Pre-harvest application of chitosan and postharvest Aloe vera gel coating enhances quality of table grape (*Vitis vinifera* L. cv. ‘Yaghouti’) during postharvest period. Food Chem..

[200] Singh R.K., Martins V., Soares B., Castro I., Falco V. (2020). Chitosan application in vineyards (*Vitis vinifera* L. cv. Tinto Cão) induces accumulation of anthocyanins and other phenolics in berries, mediated by modifications in the transcription of secondary metabolism genes. Int. J. Mol. Sci..

[201] Miliordos D.E., Tsiknia M., Kontoudakis N., Dimopoulou M., Bouyioukos C., Kotseridis Y. (2021). Impact of Application of Abscisic Acid, Benzothiadiazole and Chitosan on Berry Quality Characteristics and Plant Associated Microbial Communities of *Vitis vinifera* L. var. Mouhtaro Plants. Sustainability.

[202] Vitalini S., Ruggiero A., Rapparini F., Neri L., Tonni M., Iriti M. (2014). The application of chitosan and benzothiadiazole in vineyard (*Vitis vinifera* L. cv Groppello Gentile) changes the aromatic profile and sensory attributes of wine. Food Chem..

[203] Gutiérrez-Gamboa G., Pérez-Álvarez E.P., Rubio-Bretón P., Garde-Cerdán T. (2019). Changes on grape volatile composition through elicitation with methyl jasmonate, chitosan, and a yeast extract in Tempranillo (*Vitis vinifera* L.) grapevines. Sci. Hortic..

[204] Esparza-Reynoso S., Pelagio-Flores R., López-Bucio J. (2020). Mechanism of plant immunity triggered by Trichoderma. New and Future Developments in Microbial Biotechnology and Bioengineering.

[205] Carro-Huerga G., Compant S., Gorfer M., Cardoza R.E., Schmoll M., Gutiérrez S., Casquero P.A. (2020). Colonization of *Vitis vinifera* L. by the endophyte *Trichoderma* sp. strain T154: Biocontrol activity against *Phaeoacremonium minimum*. Front. Plant Sci..

[206] Carro-Huerga G., Mayo-Prieto S., Rodríguez-González Á., González-López Ó., Gutiérrez S., Casquero P.A. (2021). Influence of Fungicide Application and Vine Age on *Trichoderma* Diversity as Source of Biological Control Agents. Agronomy.

[207] Kamble M.V., Joshi S.M., Hadimani S., Jogaiah S. (2021). Biopriming with rhizosphere *Trichoderma harzianum* elicit protection against grapevine downy mildew disease by triggering histopathological and biochemical defense responses. Rhizosphere.

[208] Sawant I.S., Wadkar P.N., Ghule S.B., Salunkhe V.P., Chavan V., Sawant S.D. (2020). Induction of systemic resistance in grapevines against powdery mildew by *Trichoderma asperelloides* strains. Australas. Plant Pathol..

[209] Bigot G., Sivilotti P., Stecchina M., Lujan C., Freccero A., Mosetti D. (2020). Long-term effects of *Trichoderma asperellum* and *Trichoderma gamsii* on the prevention of esca in different vineyards of Northeastern Italy. Crop Prot..

[210] Lazazzara V., Vicelli B., Bueschl C., Parich A., Pertot I., Schuhmacher R., Perazzolli M. (2021). *Trichoderma* spp. volatile organic compounds protect grapevine plants by activating defense-related processes against downy mildew. Physiol. Plant..

[211] Poveda J. (2021). *Trichoderma* as biocontrol agent against pests: New uses for a mycoparasite. Biol. Control.

[212] Rodríguez-González Á., Carro-Huerga G., Mayo-Prieto S., Lorenzana A., Gutiérrez S., Peláez H.J., Casquero P.A. (2018). Investigations of *Trichoderma* spp. and *Beauveria bassiana* as biological control agent for *Xylotrechus arvicola*, a major insect pest in Spanish vineyards. J. Econ. Entomol..

[213] Azizoglu U., Yilmaz N., Simsek O., Ibal J.C., Tagele S.B., Shin J.H. (2021). The fate of plant growth-promoting rhizobacteria in soilless agriculture: Future perspectives. 3 Biotech.

[214] Sathya A., Vijayabharathi R., Gopalakrishnan S. (2017). Plant growth-promoting actinobacteria: A new strategy for enhancing sustainable production and protection of grain legumes. 3 Biotech.

[215] Rani K., Wati L. (2020). The Rhizosphere Actinobacteria and Biological Control: A Review. Environ. Ecol..

[216] Azizoglu U. (2019). Bacillus thuringiensis as a biofertilizer and biostimulator: A mini-review of the little-known plant growth-promoting properties of Bt. Curr. Microbiol..

[217] Mondello V., Songy A., Battiston E., Pinto C., Coppin C., Trotel-Aziz P., Clement C., Mugnai L., Fontaine F. (2018). Grapevine trunk diseases: A review of fifteen years of trials for their control with chemicals and biocontrol agents. Plant Dis..

[218] Laassami A., Yekkour A., Meklat A., Djemouai N., Zitouni A., Mokrane S., Lecomte P., Rey P., Berraf-Tebbal A. (2020). Actinobacteria Associated with Vineyard Soils of Algeria: Classification, Antifungal Potential Against Grapevine Trunk Pathogens and Plant Growth-Promoting Features. Curr. Microbiol..

[219] Wu H., Spagnolo A., Marivingt-Mounir C., Clément C., Fontaine F., Chollet J.F. (2020). Evaluating the combined effect of a systemic phenylpyrrole fungicide and the plant growth-promoting rhizobacteria *Paraburkholderia phytofirmans* (strain PsJN::gfp2x) against the grapevine trunk pathogen *Neofusicoccum parvum*. Pest Manag. Sci..

[220] Velásquez A., Vega-Celedón P., Fiaschi G., Agnolucci M., Avio L., Giovannetti M., D’Onofrio C., Seeger M. (2020). Responses of *Vitis vinifera* cv. Cabernet Sauvignon roots to the arbuscular mycorrhizal fungus *Funneliformis mosseae* and the plant growth-promoting rhizobacterium *Ensifer meliloti* include changes in volatile organic compounds. Mycorrhiza.

[221] Andreolli M., Zapparoli G., Lampis S., Santi C., Angelini E., Bertazzon N. (2021). In Vivo Endophytic, Rhizospheric and Epiphytic Colonization of *Vitis vinifera* by the Plant-Growth Promoting and Antifungal Strain Pseudomonas protegens MP12. Microorganisms.

[222] Torres N., Yu R., Kurtural S.K. (2021). Inoculation with Mycorrhizal Fungi and Irrigation Management Shape the Bacterial and Fungal Communities and Networks in Vineyard Soils. Microorganisms.

[223] Moukarzel R., Ridgway H.J., Guerin-Laguette A., Jones E.E. (2021). Grapevine rootstocks drive the community structure of arbuscular mycorrhizal fungi in New Zealand vineyards. J. Appl. Microbiol..

[224] Velásquez A., Valenzuela M., Carvajal M., Fiaschi G., Avio L., Giovannetti M., D’Onofrio C., Seeger M. (2020). The arbuscular mycorrhizal fungus *Funneliformis mosseae* induces changes and increases the concentration of volatile organic compounds in Vitis vinifera cv. Sangiovese leaf tissue. Plant Physiol. Biochem..

[225] Agudelo M.B., Meyer E., Lovato P.E. (2020). Growth, heavy metal uptake, and photosynthesis in ‘Paulsen 1103’ (*Vitis berlandieri* x *rupestris*) grapevine rootstocks inoculated with arbuscular mycorrhizal fungi from vineyard soils with high copper contents. Vitis J. Grapevine Res..

[226] Agudelo M.B., Meyer E., Lovato P.E. (2021). Arbuscular mycorrhizal fungus richness in the soil and root colonization in vineyards of different ages. Rhizosphere.

[227] Nogales A., Rottier E., Campos C., Victorino G., Costa J.M., Coito J.L., Pereira H.S., Viegas W., Lopes C. (2021). The effects of field inoculation of arbuscular mycorrhizal fungi through rye donor plants on grapevine performance and soil properties. Agric. Ecosyst. Environ..

[228] Massa N., Bona E., Novello G., Todeschini V., Boatti L., Mignone F., Gamalero E., Lingua G., Berta G., Cesaro P. (2020). AMF communities associated to Vitis vinifera in an Italian vineyard subjected to integrated pest management at two different phenological stages. Sci. Rep..

[229] Landi L., Foglia R., Murolo S., Romanazzi G. (2021). The Mycorrizal Status in Vineyards Affected by Esca. J. Fungi.

[230] Etesami H. (2018). Can interaction between silicon and plant growth promoting rhizobacteria benefit in alleviating abiotic and biotic stresses in crop plants?. Agric. Ecosyst. Environ..

[231] Bakhat H.F., Bibi N., Zia Z., Abbas S., Hammad H.M., Fahad S., Ashraf M.R., Shah G.M., Rabbani F., Saeed S. (2018). Silicon mitigates biotic stresses in crop plants: A review. Crop Prot..

[232] Rajput V.D., Minkina T., Feizi M., Kumari A., Khan M., Mandzhieva S., Sushkova S., El-Ramady H., Verma K.K., Singh A. (2021). Effects of silicon and silicon-based nanoparticles on rhizosphere microbiome, plant stress and growth. Biology.

[233] Schabl P., Gabler C., Kührer E., Wenzel W. (2020). Effects of silicon amendments on grapevine, soil and wine. Plant Soil Environ..

[234] Amato D., Montanaro G., Summerer S., Briglia N., Attia F., Challet E., Nuzzo V. (2020). The effects of calcite silicon-mediated particle film application on leaf temperature and grape composition of Merlot (*Vitis vinifera* L.) vines under different irrigation conditions: This article is published in cooperation with the XIIIth International Terroir Congress November 17–18 2020, Adelaide, Australia. Guest editors: Cassandra Collins and Roberta De Bei. OENO One.

[235] Farouk S., Belal B.E.A., El-Sharkawy H.H.A. (2017). The role of some elicitors on the management of Roumy Ahmar grapevines downy mildew disease and it’s related to inducing growth and yield characters. Sci. Hortic..

[236] Farouk S., El-Metwally I.M. (2019). Synergistic responses of drip-irrigated wheat crop to chitosan and/or silicon under different irrigation regimes. Agric. Water Manag..

[237] Habibi G. (2015). Effects of soil-and foliar-applied silicon on the resistance of grapevine plants to freezing stress. Acta Biol. Szeged..

[238] Qin L., Kang W.H., Qi Y.L., Zhang Z.W., Wang N. (2016). The influence of silicon application on growth and photosynthesis response of salt stressed grapevines (*Vitis vinifera* L.). Acta Physiol. Plant..

[239] Aguín O., Mansilla J.P., Sainz M.J. (2006). In vitro selection of an effective fungicide against *Armillaria mellea* and control of white root rot of grapevine in the field. Pest Manag. Sci. Former. Pestic. Sci..

[240] Pereira V.F., Resende M.L.V.D., Ribeiro Junior P.M., Regina M.D.A., Mota R.V.D., Vitorino L.R.R. (2012). Potassium phosphite on the control of downy mildew of grapevine and physicochemical characteristics of Merlot grapes. Pesqui. Agropecuária Bras..

[241] Pinto K.M.S., do Nascimento L.C., de Souza Gomes E.C., da Silva H.F., dos Reis Miranda J. (2012). Efficiency of resistance elicitors in the management of grapevine downy mildew *Plasmopara viticola*: Epidemiological, biochemical and economic aspects. Eur. J. Plant Pathol..

[242] Buffara C.R.S., Angelotti F., Tessmann D.J., de Souza C.D., Vida J.B. (2013). Potassium phosphite pre-and post-infection activities against *Phakopsora euvitis* in grapevine leaves. Semin. Ciências Agrárias.

[243] Da Silva H.F., Pinto K.M.S., do Nascimento L.C., da Silva E.C., de Souza W.C.O. (2019). Evaluation of the use of biotic and abiotic resistance elicitors against anthracnose in grapevine (*Vitis labrusca* L.). Summa Phytopathol..

[244] Burdziej A., Bellée A., Bodin E., Valls Fonayet J., Magnin N., Szakiel A., Richard T., Cluzet S., Corio-Costet M.F. (2021). Three types of elicitors induce grapevine resistance against downy mildew via common and specific immune responses. J. Agric. Food Chem..

